# An Enhanced Secretary Bird Optimization Algorithm Based on Multi Population Management for Numerical Optimization Problems

**DOI:** 10.3390/biomimetics10110761

**Published:** 2025-11-12

**Authors:** Jin Zhu, Bojun Liu, Jun Zheng, Shaojie Yin, Meng Wang

**Affiliations:** 1 School of Journalism and Communication, Tsinghua University, Beijing 100000, China; zhengce360@vip.163.com; 2 Faculty of Engineering, The University of Sydney, Sydney, NSW 2006, Australia; liubojun9999@gmail.com; 3 Taizhou Institute of Zhejiang University, Taizhou 318000, China; yinshaojie@tzizju.cn

**Keywords:** secretary bird optimization algorithm, swarm intelligence, multi-population management, experience trend guiding, benchmark test suite

## Abstract

The Secretary Bird Optimization Algorithm (SBOA) is a novel swarm-based meta-heuristic that formulates an optimization model by mimicking the secretary bird’s hunting and predator-evasion behaviors, and thus possesses appreciable application potential. Nevertheless, it suffers from an unbalanced exploration–exploitation ratio, difficulty in maintaining population diversity, and a tendency to be trapped in local optima. To eliminate these drawbacks, this paper proposes an SBOA variant (MESBOA) that integrates a multi-population management strategy with an experience-trend guidance strategy. The proposed method is compared with eight advanced basic/enhanced algorithms of different categories on both the CEC2017 and CEC2022 test suites. Experimental results demonstrate that MESBOA delivers faster convergence, more stable robustness and higher accuracy, achieving mean rankings of 2.500 (CEC2022 10-D), 2.333 (CEC2022 20-D), 1.828 (CEC2017 50-D) and 1.931 (CEC2017 100-D). Moreover, engineering constrained optimization problems further verify its applicability to real-world optimization tasks.

## 1. Introduction

Global optimization problems are ubiquitous in modern complex systems and engineering design [1,2]. They are typically characterized by high non-linearity, multimodality, multiple constraints and large dimensionality, posing significant challenges to traditional optimization techniques [3]. Classical deterministic approaches such as gradient-based methods and dynamic programming perform well on simple tasks like convex optimization, but often fail to provide satisfactory solutions when the problem is non-convex or non-differentiable [4]. In contrast, meta-heuristic methods, which operate without gradient information, can effectively explore the search space and have been widely applied to complex global optimization and engineering design tasks [5]. Although these algorithms do not guarantee global optimality, they are highly valuable because they consistently deliver high-quality solutions for large-scale and intricate problems [6]. Numerous studies have demonstrated their superior performance in practical engineering challenges ranging from mechanical structure design and parameter estimation to mission planning [7,8,9,10,11]. However, as problem size and complexity grow, the performance of meta-heuristics may degrade markedly, making the continuous development and refinement of new algorithms an urgent necessity [12].

The central idea of meta-heuristic algorithms is to design adaptive and robust search strategies by emulating natural phenomena, biological behaviors, or physical laws. Representative categories and algorithms are briefly exemplified below [13].

Evolutionary-based algorithms realize optimization by replicating the “selection–crossover–mutation” cycle observed in biological evolution. For example, the canonical genetic algorithm (GA) [14] iteratively refines a population through encoded individuals, fitness evaluation, and the genetic operators of selection, crossover, and mutation. Differential evolution (DE) [15] creates candidate solutions by adding scaled difference vectors between existing individuals, a mechanism that is particularly effective in continuous search spaces. Other representatives of this family include Evolution Strategies (ES) [16] and the recently proposed Alpha Evolution (AE) [17], all of which follow the same evolutionary paradigm while introducing distinct variation and selection schemes.

Physics-based algorithms abstract optimization rules from the laws of physics or chemical reactions observed in nature. Inspired by the annealing of metals, Simulated Annealing (SA) [18] employs a temperature schedule to probabilistically accept inferior solutions, thereby escaping local optima. The Gravitational Search Algorithm (GSA) [19] models candidate solutions as masses that interact through Newtonian gravity; heavier masses (better solutions) attract others, guiding the population toward high-fitness regions. Other representatives of this category include Polar Lights Optimization (PLO) [20], Fick’s Law Algorithm (FLA) [21], Light Spectrum Optimizer (LSO) [22] and Fata Morgana Algorithm (FMA) [23].

Human-based algorithms distill optimization logic from human activities and social collaboration. The Teaching–Learning-Based Optimization (TLBO) [24], for instance, mimics the knowledge-transfer process between an instructor and learners in a classroom, whereas the Social Group Optimization (SGO) [25] exploits the cooperative mechanisms of information sharing and collective decision-making observed in human social networks. Other algorithms that belong to this category include the Political Optimizer (PO) [26], Football Team Training Algorithm (FTTA) [27], Escape Optimization Algorithm (EOA) [28], Catch Fish Optimization Algorithm (CFOA) [29], and Student Psychology Based Optimization (STBO) [30].

Swarm-based algorithms mimic the cooperative behavior of biological swarms and achieve global optimization through information sharing among simple agents. Particle Swarm Optimization (PSO) [31], for instance, emulates the foraging of bird flocks: each particle adjusts its position and velocity at every iteration by simultaneously learning from its personal best experience and the swarm’s global best, rapidly converging toward promising regions. Ant Colony Optimization (ACO) [32] replicates the pheromone trail communication of foraging ants; artificial ants probabilistically construct paths and reinforce shorter ones through pheromone updates, eventually converging to minimal routes. Grey Wolf Optimizer (GWO) [33] abstracts the strict social hierarchy and collective hunting tactics of grey wolves—tracking, encircling, and attacking prey—to balance exploration and exploitation in multimodal landscapes. Beyond these mainstream methods, a growing family of swarm intelligence techniques—such as Greylag Goose Optimization (GGO) [34], Tuna Swarm Optimization (TSO) [35], Sled Dog Optimizer (SDO) [36], and Crayfish Optimization Algorithm (COA) [37]—continues to emerge, offering alternative neighborhood topologies and communication rules for diverse optimization scenarios.

For meta-heuristic algorithms, exploration and exploitation constitute the two core phases that directly govern global search capacity and local refinement efficiency; balancing them is therefore pivotal to overall performance [38]. Exploration refers to the extensive sampling of the search space to identify promising regions, thereby reducing the risk of premature entrapment in local optima and safeguarding the discovery of the global solution. Exploitation, in contrast, concentrates the search within these promising regions to refine solutions, improving accuracy and accelerating convergence. Unfortunately, the foundational variants of most meta-heuristics suffer from inherent bottlenecks—premature convergence, local-optimum stagnation, and slow convergence—when confronted with complex problems. To alleviate these drawbacks, researchers have proposed novel operators or refined existing ones to strengthen both global exploration and local exploitation capabilities. In parallel, hybrid algorithms that synergize operators drawn from different meta-heuristics have been developed to achieve an adaptive equilibrium between the two search modes. Reinforcing this trend, the No-Free-Lunch theorem [39] asserts that no single algorithm can outperform all others across every optimization problem, continuously motivating the emergence of new intelligent optimization paradigms.

The SBOA is a novel swarm-based meta-heuristic that emulates the secretary bird’s hunting and escape tactics. The optimization process is explicitly divided into an exploration phase and an exploitation phase, each governed by distinct search behaviors executed in sequence. Owing to these diversified strategies, SBOA delivers competitive performance on a variety of benchmark functions and engineering cases. In the original study by Fu et al., SBOA outperformed basic algorithms such as WOA, GWO, COA and DBO, and achieved results comparable with the advanced LSHADE-SPACMA [40]. Although the original SBOA has shown certain merits, follow-up studies reveal that it still suffers from several drawbacks when confronted with complex optimization scenarios, such as the following: local optimum stagnation—on high-dimensional, multimodal or irregular landscapes the population often stalls around sub-optimal regions and is unable to jump out [41]; limited global exploration—in the early search stage the diversity of the swarm is frequently insufficient, so the algorithm cannot thoroughly cover the solution space [42]; premature convergence—blind attraction toward the current global best easily misguides the whole population into a local optimum, after which further improvement becomes difficult [43]. Consequently, enhancing the global exploration ability and the exploitation accuracy—while simultaneously avoiding local optima—has become the focus of recent research. To mitigate these drawbacks, several SBOA variants have recently been proposed. Xu et al. enriched population diversity through an adaptive learning strategy and balanced exploration and exploitation with a multi-population evolution scheme [44]. Zhu et al. embedded a Student’s t-distribution mutation derived from quantum computing to help the algorithm escape local optima [45]. Song et al. accelerated exploitation by introducing a golden-sine guidance operator while preserving diversity with a cooperative camouflage mechanism [46]. Meng et al. reduced the risk of stagnation via a differential cooperative search and speeded up convergence with an information-retention control strategy [47]. Although these variants improve performance, most of them retain the original SBOA framework in which exploration and exploitation are executed simultaneously within a single iteration. This synchronous update can still lead to incomplete search and premature convergence. Moreover, the extra operators often raise algorithmic complexity and computational cost, creating new challenges that remain to be addressed.

To mitigate the inherent limitations of the original SBOA, this paper proposes an enhanced variant, MESBOA, which incorporates a multi-population management strategy and an experience-trend guidance strategy. The main contributions are summarized as follows:

(1) Dual-mechanism bottleneck relief. A new multi-population management protocol restructures the SBOA framework so that exploration and exploitation are executed in separate subpopulations, eliminating mutual interference and guaranteeing a balanced search. An experience-trend guidance operator dynamically extracts historical information from elite groups and uses it to steer the entire swarm along the most promising evolutionary direction. Acting synergistically, these two mechanisms enlarge global exploration breadth, refine local exploitation accuracy, and reinforce convergence robustness;
(2) Comprehensive benchmark validation. MESBOA is systematically compared with several state-of-the-art basic and improved algorithms on both the 10/20-D CEC-2022 and the 50/100-D CEC-2017 test suites. Statistical analyses—including Friedman, Wilcoxon rank-sum and Nemenyi tests—consistently rank MESBOA among the top performers;
(3) Superior performance on real-world engineering tasks. When applied to a variety of constrained mechanical-design problems, MESBOA reliably obtains better feasible solutions than its competitors while exhibiting markedly lower variance, offering practitioners an efficient and stable optimization tool, especially for high-dimensional complex systems.

This paper is organized as follows: Section 2 presents primarily knowledge of the Secretary Bird Optimization Algorithm. The proposed MPSBOA method with exploration and exploitation strategies is explained in Section 3. Experimental studies and the results are presented in Section 4 and Section 5. Finally, the conclusion is given in Section 6.

## 2. Secretary Bird Optimization Algorithm

The Secretary Bird Optimization Algorithm (SBOA) translates the bird’s survival strategies—predation and predator avoidance—into a three-stage search template, with initialization, exploration, and exploitation. The exploration phase models hunting behavior and is subdivided into (i) prey search, (ii) prey exhausting, and (iii) prey attack. The exploitation phase mirrors anti-predator behavior, i.e., camouflage or flight. Detailed mathematical models of each stage are presented below.

### 2.1. Initialization Phase

In SBOA, each secretary bird corresponds to one member of the swarm; its position vector in the search space encodes the decision variables and therefore represents a single candidate solution. As a swarm-based algorithm, SBOA adopts the conventional initialization, where every bird is randomly placed inside the variable bounds using Equation (1).

(1) $$X_{i}^{0}=lb+\left(ub-lb\right)\times rand\left(1,D\right)$$

where $X_{i}^{0}$ is the initial value of the *i*th candidate solution. lb and ub are the lower and upper bound vectors of the search space, respectively. $rand\left(1,D\right)$ is a random vector with elements uniformly distributed in [0, 1]. By applying the above formula to all individuals in the population, the initialized population can be represented as

(2) $$X=\left[\begin{array}{c} X_{1}\\ X_{2}\\ \vdots \\ X_{i}\\ \vdots \\ X_{N} \end{array}\right]=\left[\begin{array}{cccccc} x_{1,1} & x_{1,2} & \ldots & x_{1,j} & \ldots & x_{1,D}\\ x_{2,1} & x_{2,2} & \ldots & x_{2,j} & \ldots & x_{2,D}\\ \vdots & \vdots & \ddots & \vdots & \ddots & \vdots \\ x_{i,1} & x_{i,2} & \ldots & x_{i,j} & \ldots & x_{i,D}\\ \vdots & \vdots & \ddots & \vdots & \ddots & \vdots \\ x_{N,1} & x_{N,2} & \ldots & x_{N,j} & \ldots & x_{N,D} \end{array}\right]$$

where N denotes the population size and D denotes the dimension of the problem. After initialization, the fitness value of each individual is evaluated, and the one with the highest fitness is selected as the current global best solution.

### 2.2. Exploration Phase

The exploration phase of SBOA consists of three sequential search processes—prey searching, prey exhausting, and prey attacking—which are activated in strict order as the run proceeds.

During the first third of the iterations only the prey-search model is executed; it is then replaced by the prey-exhaust model in the middle third, and finally the prey-attack model is applied during the last third of the iterations. The three exploration behaviors are detailed next.

During the prey-search phase (*t* < 1/3 *T*), SBOA adjusts the position of every secretary bird by exploiting inter-individual differences, thereby enhancing population diversity and global exploration. The corresponding mathematical model is given by Equation (3).

(3) $$X_{i}^{new}=X_{i}+\left(X_{r1}-X_{r2}\right)\times rand\left(1,D\right)$$

where $X_{i}^{new}$ is the updated position of the *i*th secretary bird agent. $X_{r1}$ and $X_{r2}$ are two distinct individuals randomly selected from the population. During the prey-exhaust phase (1/3 *T* < *t* < 2/3 *T*), SBOA models the random walk of the secretary bird by Brownian motion, as expressed in Equation (4).

(4) $$X_{i}^{new}=X_{best}+e^{{\left(\frac{t}{T}\right)}^{4}}\times \left(RB-0.5\right)\times \left(X_{best}-X_{i}\right)$$

where t and T denote the current iteration number and the maximum iteration number, respectively. $X_{best}$ is the global best individual. e is the natural constant, and RB is a random vector drawn from the standard normal distribution. During the final prey-attack phase (*t* > 2/3 *T*), the secretary bird employs Lévy flight to mimic its striking mobility, as described by Equation (5).

(5) $$X_{i}^{new}=X_{best}+{\left(1-\frac{t}{T}\right)}^{\frac{2t}{T}}\times X_{i}\times RL$$

where RL is a random vector drawn from the Lévy distribution, as given by Equation (6).

(6) $$RL=0.5\times L\mathrm{evy}\left(D\right)$$

(7) $$L\mathrm{evy}\left(D\right)=0.01\times \frac{\mu \times \sigma }{{\left|v\right|}^{\frac{1}{\eta }}}$$

Here, η is a fixed constant of 1.5. μ and σ are random numbers in the interval [0, 1]. The formula for *σ* is as follows:

(8) $$\sigma ={\left(\frac{\Gamma \left(1+\eta \right)\times \sin\left(\frac{\pi \eta }{2}\right)}{\Gamma \left(\frac{1+\eta }{2}\right)\times \eta \times 2\left(\frac{\eta -1}{2}\right)}\right)}^{\frac{1}{\eta }}$$

where $\Gamma \left(\cdot \right)$ denotes the gamma function.

### 2.3. Exploitation Phase

When evading predators, the secretary bird adopts two tactics—flight or camouflage. SBOA translates these tactics into the exploitation-phase model; each behavior is executed with equal probability, as expressed in Equations (9) and (10).

(9) $$X_{i}^{new}=X_{best}+\left(2\times RB-1\right)\times {\left(1-\frac{t}{T}\right)}^{2}\times X_{i}$$

(10) $$X_{i}^{new}=X_{i}+R_{1}\times \left(X_{r3}-K\times X_{i}\right)$$

where $R_{1}$ is a random vector drawn from the standard normal distribution. $X_{r3}$ is an individual randomly selected from the secretary bird population, and K is a random integer equal to either 1 or 2.

## 3. The Proposed MESBOA

This section presents MESBOA and devises two enhancement strategies to overcome the original SBOA’s imbalance between exploitation and exploration and its tendency to fall into local optima.

### 3.1. Multi Population Management Strategy

For meta-heuristics, exploration features wide search coverage, high diversity and strong randomness, and is therefore preferred in early iterations, multimodal landscapes or dynamic environments. Exploitation, by contrast, is characterized by narrow search range, increased determinism and rapid convergence, so it is better suited to later iterations, unimodal problems or static environments.

The basic SBOA, however, executes the exploration and exploitation behaviors in sequence within every single iteration: each bird first performs an exploratory move and then immediately applies an exploitative update to the same individual. This updating pattern has two defects. Excessive exploitation in early stages wastes evaluations on unpromising regions and restricts the search breadth. Excessive exploration in later stages prevents the swarm from focusing on the most promising areas and slows convergence. The multi-population (or sub-population) strategy is a well-established and widely adopted enhancement technique in the field of meta-heuristics. By dividing the entire search workforce into several mutually interacting sub-groups and equipping each sub-group with its own search operator, the algorithm is able to maintain higher diversity, explore different regions of the search space in parallel, and thus achieve a better balance between exploration and exploitation. This idea has been successfully integrated into numerous optimization algorithms—ranging from differential evolution and particle swarm optimization to artificial bee colony and genetic algorithms—yielding consistent performance improvements across a broad set of benchmark and real-world problems [48,49,50]. To overcome these drawbacks, we propose a multi-population management strategy (MMS) that restructures the SBOA search framework and coordinates exploration and exploitation instead of running them back-to-back. Specifically, MMS splits the population into three sub-groups—dominant, balanced and inferior—according to fitness.

The dominant group act as leaders. In early stages they enlarge the search scope to guarantee strong global exploration, while in later stages they retain the ability to jump out of local optima and thus avoid premature convergence.

The balanced group are responsible for a smooth transition between exploration and exploitation, ensuring the algorithm keeps refining the solution without oscillation.

Regarding the inferior group, although they are far from the optimum, they are not discarded. Opposition-based learning suggests that the opposite of a poor solution may be promising; hence these individuals continue to broaden the search and, in later stages, can accelerate convergence by learning from the best.

Based on the above analysis, MMS reallocates the original SBOA operators so that exploration and exploitation are executed by different sub-populations at the same time, eliminating mutual interference and achieving a cooperative balance.

MMS assigns Equations (2) and (5) to the dominant group. When rand<t/T is satisfied, Equation (5) is executed for position updating; otherwise, Equation (2) is applied. This switching guarantees intensive global exploration at the beginning while retaining the ability to jump out of local optima, and shifts the emphasis to deep exploitation in later stages while still preserving diversity. The balanced group is updated by the secretary bird’s escape and camouflage behaviors. If rand<t/T, Equation (10) is used; otherwise, Equation (9) is performed.

This mechanism keeps the balanced individuals moving toward the current best while periodically enlarging their search radius, thereby sustaining an equilibrium between exploration and exploitation. For the inferior group, the prey-search and prey-exhaust operators are adopted. When rand<t/T is met, Equation (2) is executed; otherwise, Equation (4) is employed. Consequently, the inferior individuals continuously broaden the search scope and, by learning from the best in the final phase, accelerate convergence without misleading the rest of the swarm. The pseudo-code of MMS is given in Algorithm 1.
**Algorithm 1**: Pseudocode of multi-population management strategy (MMS)1: Input: *lb*, *ub*, *D*, *N*, *T*2: Initialize population randomly according to Equation (1)3: While (*t* < *T*) do4:   Calculate the fitness of each secretary bird individual5:   For *i* =1: *N*6:     If *X_i_* belongs to dominant group7:       If rand<t/T
8:          Update the position of secretary bird individual using Equation (5)9:       Else10:          Update the position of secretary bird individual using Equation (2)11:       End if12:     Else if *X_i_* belongs to balanced group13:       If rand<t/T
14:          Update the position of secretary bird individual using Equation (10)15:        Else16:          Update the position of secretary bird individual using Equation (9)17:       End if18:     Else19:       If rand<t/T
20:          Update the position of secretary bird individual using Equation (2)21:        Else22:          Update the position of secretary bird individual using Equation (4)23:       End if24:     End if25:  End for26: *t* = *t* + 127: End while28: Output: The best solution *X_b_*

### 3.2. Experience-Trend Guidance Strategy

SBOA neglects inter-individual information exchange; consequently, population diversity collapses in the later search period. Moreover, its limited global exploration capacity and insufficient local refinement ability restrict overall search performance. The algorithm also discards the historical data accumulated during iterations, so it cannot fully capture latent search dynamics or trends. The guided learning strategy is an experience-driven enhancement technique that evaluates the instantaneous exploration–exploitation demand from the collective search history of all individuals and then adaptively selects the most suitable search mode; however, because the decision is based on the averaged past experience of the whole swarm, it may mistake a temporary local aggregation for a genuine convergence signal and consequently mistrigger excessive exploitation, leading to erroneous guidance and degraded global exploration capability [51]. To remedy these weaknesses we propose an experience-trend guidance strategy (EGS) that exploits the historical information of dominant individuals to steer the optimization process.

Specifically, EGS computes the standard deviation of recent positions to measure population dispersion and infers the type of guidance currently required. When the algorithm is biased toward exploration, EGS switches the search to exploitation; otherwise, it drives the swarm back toward exploration. Meanwhile, by analyzing the positional records of dominant individuals over previous iterations, EGS extracts the evolutionary trend and locates regions that are likely to contain the global optimum. In this way, EGS dynamically alternates between guiding exploitation and exploration according to the instantaneous search state, achieving an effective exploration–exploitation balance. The mathematical model of EGS is given below.

**Figure 1:**
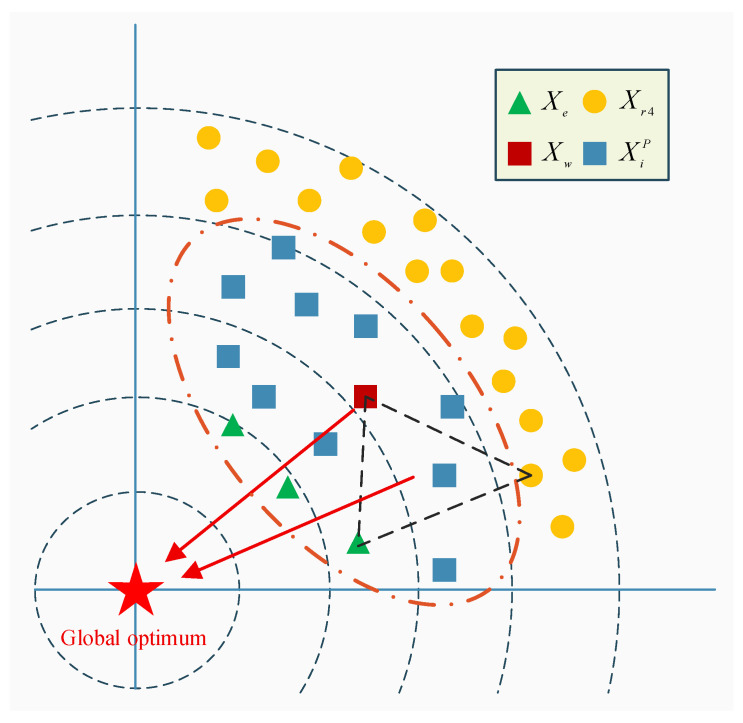
The schematic of EGS.

First, every individual of each iteration is stored in a historical memory pool P whose maximum capacity is Pmax. When the number of stored individuals exceeds Pmax, the standard deviation of these archived individuals is computed with Equation (11), and the resulting value is normalized by B obtained from Equation (12) to eliminate sensitivity to variable-bound changes.

(11) $$V_{0}=std\left(P\right)\times B$$

(12) $$B=200/\left(lb-ub\right)$$

where $std\left(\cdot \right)$ is the function to calculate standard deviation. After obtaining $V_{0}$, EGS updates individuals through two adaptive schemes: an exploration-oriented update where, if $V_{0}$ indicates low population diversity (i.e., the swarm is highly clustered), EGS relocates individuals toward under-explored regions via Equation (14) to prevent premature convergence and sustain exploration momentum, and an exploitation-oriented update where, if $V_{0}$ exceeds the threshold (i.e., the swarm is overly dispersed), EGS triggers an exploitation operator via Equation (13) that intensifies the search around promising areas through the combined influence of the elite group, the global best agent and a randomly chosen agent to accelerate convergence, with the switch between these two modes being self-adaptive to guarantee a dynamic balance between exploration and exploitation according to the evolutionary state inferred from $V_{0}$.

(13) $$X_{i}^{new}=\frac{\left(X_{r4}+X_{w}+X_{e}\right)}{3}+g_{i},g_{i}\sim N\left(0,Cov\right),\;if\;V_{0}>c$$

(14) $$X_{i}^{new}=X_{w}+g_{i},g_{i}\sim N\left(0,Cov\right),if\;V_{0}\leq c$$

(15) $$Cov=\frac{1}{\left|P_{d}\right|}\sum_{i=1}^{\left|P_{d}\right|}\left(X_{i}^{P}-X_{w}\right)\times {\left(X_{i}^{P}-X_{w}\right)}^{T},\;X_{i}^{P}\in P_{d}$$

(16) $$X_{w}=\sum_{i=1}^{\left|P_{d}\right|}\omega_{i}\times X_{i}^{P},X_{i}^{P}\in P_{d}$$

(17) $$\omega_{i}=\ln\left(\left|P_{d}\right|+1\right)/\left(\sum_{i=1}^{\left|P_{d}\right|}\left(\ln\left(\left|P_{d}\right|+1\right)-\ln\left(i\right)\right)\right)$$

where $X_{r4}$ is a secretary bird individual randomly selected from the current population. $X_{w}$ denotes the weighted average position of the dominant individuals stored in the historical memory pool P. $X_{e}$ is a randomly chosen individual among the top three fittest birds in the current swarm. Cov is the covariance matrix of the dominant population. $P_{d}$ represents the dominant individuals preserved in P, and $\left|P_{d}\right|$ gives the number of such dominant individuals. As illustrated in Figure 1, the dominant group drives the population toward promising regions, the top three randomly chosen individuals supply alternative directions while accelerating convergence, and the random agent enlarges the set of possible search orientations; consequently Equation (14) markedly improves individual quality and strengthens global exploration, whereas Equation (13) speeds up convergence yet still preserves the possibility of correcting the search direction.

### 3.3. Implementation Steps of MESBOA

**Figure 2:**
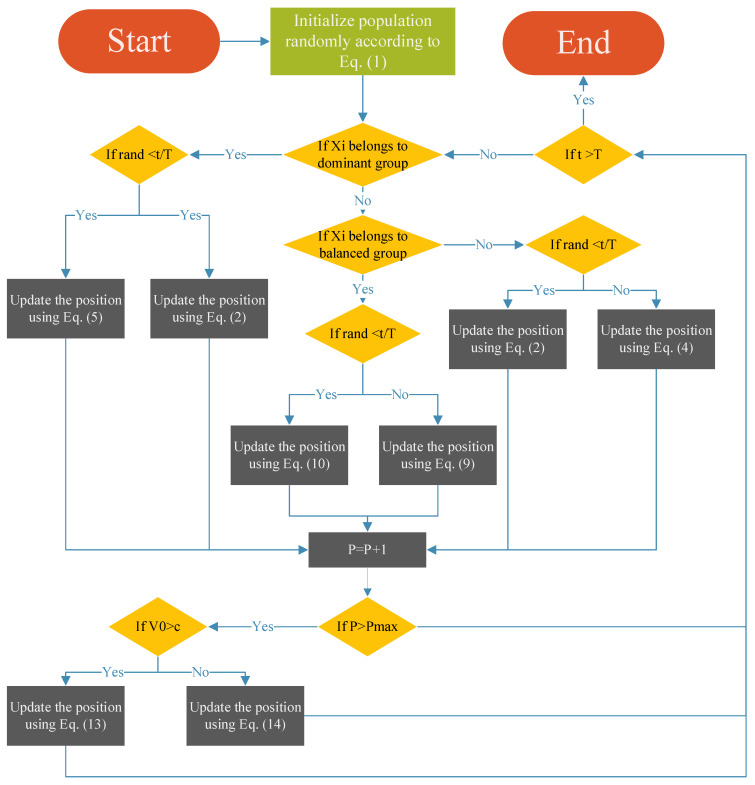
Flowchart of the proposed MESBOA.

In summary, the proposed MESBOA first generates an initial set of solutions within the search bounds via Equation (1). During each iteration, every individual is updated by the search operator designated by MMS; the renewed individuals are then saved in pool *P*. Once the EGS activation condition is met, each dimension of every individual is revised by either Equation (13) or Equation (14) according to the current demand. This loop repeats until the stopping criterion is satisfied. The pseudo-code of MESBOA is given in Algorithm 2 and its flowchart is illustrated in Figure 2.
**Algorithm 2**: Pseudocode of MESBOA1: Input: *lb*, *ub*, *D*, *N*, *T*2: Initialize population randomly according to Equation (1)3: While (*t* < *T*) do4:   Calculate the fitness of each secretary bird individual5:   For *i* = 1: *N*6:     If *X_i_* belongs to dominant group7:       If rand<t/T
8:          Update the position of secretary bird individual using Equation (5)9:       Else10:          Update the position of secretary bird individual using Equation (2)11:       End if12:     Else if *X_i_* belongs to balanced group13:       If rand<t/T
14:          Update the position of secretary bird individual using Equation (10)15:        Else16:          Update the position of secretary bird individual using Equation (9)17:       End if18:     Else19:       If rand<t/T
20:          Update the position of secretary bird individual using Equation (2)21:        Else22:          Update the position of secretary bird individual using Equation (4)23:       End if24:     End if25:     P=P+1
26:      If P≥Pmax
27:       If $V_{0}>c$
28:             Update the position of secretary bird individual using Equation (13)29:       Else30:             Update the position of secretary bird individual using Equation (14)31:       End if32:     End if33:  End for34: *t* = *t* + 135: End while36: Output: The best solution *X_b_*

### 3.4. The Computational Complexity of MESBOA

Time complexity is a critical metric for evaluating the performance of optimization algorithms. The computational cost of both SBOA and MESBOA is dominated by population initialization and iterative updating, with the key factors being the maximum number of iterations (T), problem dimension (D), and population size (N). According to the original SBOA paper, its time complexity is $O\left(2T\times N\times D\right)$. Below is the time complexity of MESBOA. The time complexity of initializing the secret birds population is $O\left(N\times D\right)$. In the position-update stage, MMS only reassigns the existing search operators; each individual is still moved once per iteration. Hence, the time complexity of MMS remains $O\left(T\times N\times D\right)$. As an extra position-update module, GES adds its own cost to SBOA. If EGS is invoked $T_{1}$ times, its time complexity is $O\left(T_{1}\times N\times D\right)$. Therefore, the overall time complexity of MESBOA is $O\left(\left(T_{1}+T\right)\times N\times D\right)$. Since the EGS procedure is triggered only after the archive has reached its maximum capacity Pmax, we assumed Pmax is $N_{1}\times D$. Consequently, EGS will be executed $T/N_{1}$ times, yielding $T_{1}=T/N_{1}$, and because $N_{1}>1$, it follows immediately that *T_1_* < *T*. Thus, the overall time complexity of MESBOA is slightly lower than that of the original SBOA.

## 4. Benchmark Test Results and Analysis

To comprehensively evaluate the performance of the proposed MESBOA, a total of 41 functions from the CEC2017 and CEC2022 test suites are employed. In this section, we conduct parameter sensitivity analysis, ablation experiments, convergence analysis, robustness analysis and statistical tests to demonstrate the superiority of MESBOA, and compare its results with various baseline and improved algorithms of different types. The section is organized into six parts: benchmark test function, experimental setup and competitor algorithms, parameter sensitivity analysis, an ablation study, low-dimensional experiments and high-dimensional experiments, which will be presented in sequence.

### 4.1. Review the Benchmark Test Suites

Two benchmark suites are employed in the experiments. The CEC 2017 set consists of unimodal (UM—F1, F3; F2 was officially removed), multimodal (MM—F4–F10), hybrid (H—F11–F20), and composite (C—F21–F30) functions. The CEC 2022 suite contains unimodal (UM—F1), multimodal (MM—F2–F5), hybrid (H—F6–F8), and composite (C—F9–F12) functions. While CEC 2017 can be evaluated at 10 D, 30 D, 50 D and 100 D, and CEC 2022 at 10 D and 20 D, we selected 50 D and 100 D cases from CEC 2017 together with 10 D and 20 D cases from CEC 2022 to examine MESBOA across a broad dimensional range. Detailed specifications for both suites are summarized in Table A1 and Table A2 of Appendix A.

### 4.2. Experimental Environment and Configuration

The experiments are realized on MATLAB 2021b in a Windows 11 environment. The platform has 32 GB RAM and an AMD R9 7945HX processor. To guarantee fairness and persuasiveness, all algorithms compared in the performance tests were run on identical data sets, the maximum number of function evaluations was fixed at 1000 D, and every algorithm was executed over 30 independent trials; to reduce randomness, the minimum value (Min), average value (Avg), and standard deviation (Std) of each metric across the 30 runs was used for statistical analysis. Statistical significance was examined via Wilcoxon rank-sum test, Friedman test and Nemenyi test.

To highlight the superiority of the proposed MESBOA, eight basic and enhanced algorithms of distinct categories are selected for comparison. These include evolution-based AE [17] and LSHADE-SPACMA [52], physics-based EO [53] and GLS-RIME [51], human-based CFOA [29] and ISGTOA [54], and swarm-based RBMO [55] and ESLPSO [56]. The AE, CFOA and RBMO are recently published high-performance basic algorithms, EO is a widely cited method, LSHADE-SPACMA is an advanced differential-evolution variant, ESLPSO represents the latest upgrade of the classical PSO, while GLS-RIME and ISGTOA have demonstrated strong efficacy in their respective studies. Overall, this diverse set of competitors provides a solid basis for verifying the exceptional performance of MESBOA. Parameter values for all contenders are taken from their original papers; only the common termination criterion (maximum function evaluations) is imposed to ensure a fair comparison. Table 1 outlines the specific parameter settings.

### 4.3. Parameter-Sensitivity Analysis

For meta-heuristic algorithms, appropriate parameter values are essential to fully exploit their potential. The proposed MESBOA integrates MMS and EGS, each of which introduces its own tunable parameters; therefore, this subsection is devoted to identifying the best settings for both modules.

**Figure 3:**
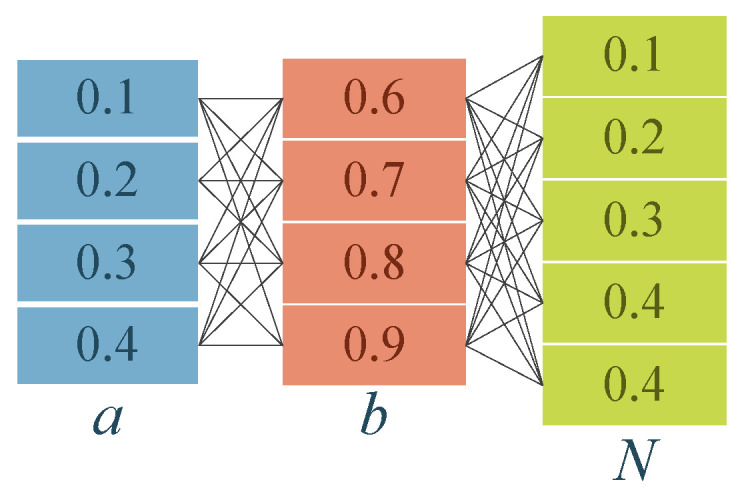
Combination of three fine-tuning parameters.

The MMS partitions the population into three groups to boost SBOA, so fixing the sizes of these groups is critical, and a grid search is therefore adopted in which the dominant group is set to contain a×N individuals, the inferior group $\left(1-b\right)\times N$, and the total population remains N, with a sweep from 0.1 to 0.4 in steps of 0.1, b from 0.6 to 0.9 in steps of 0.1, and N from 5D to 30D in steps of 5D, yielding 96 combinations (Figure 3); the version of MESBOA that employs only MMS is run 30 times on selected low- and high-dimensional functions for every parameter triple, and the results are analyzed with the Friedman test to identify the best configuration.

**Figure 4:**
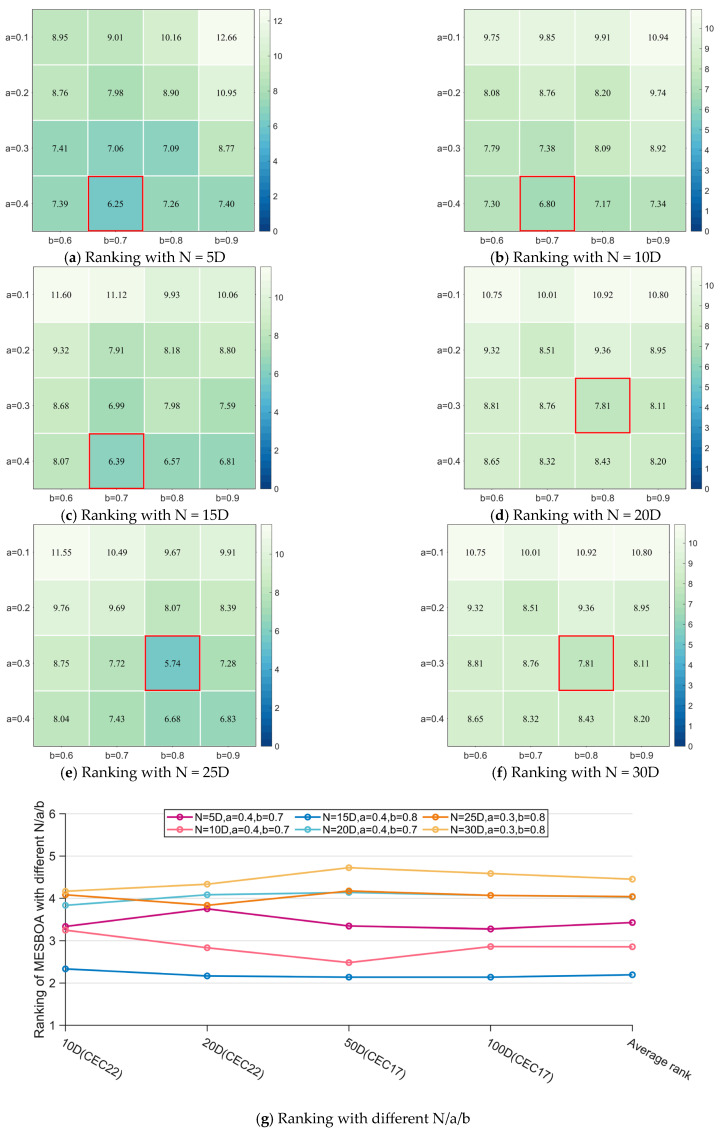
The Friedman test results of MESBOA with different N/a/b.

Figure 4 visually presents the Friedman mean ranks of MESBOA under different parameter settings for the four-dimensional cases of the two test suites; examining Figure 4a–f reveals that the algorithm achieves the top rank when a is 0.3 or 0.4 and b is 0.7 or 0.8, indicating that these parameters are insensitive to overall population size. A small a (dominant group too small) degrades performance, whereas b (inferior group) must be neither too large nor too small, confirming its constructive role. Figure 4g further shows that the best overall rank occurs at N = 15 D with a = 0.4 and b = 0.8, so this parameter set is adopted in all subsequent experiments.

**Figure 5:**
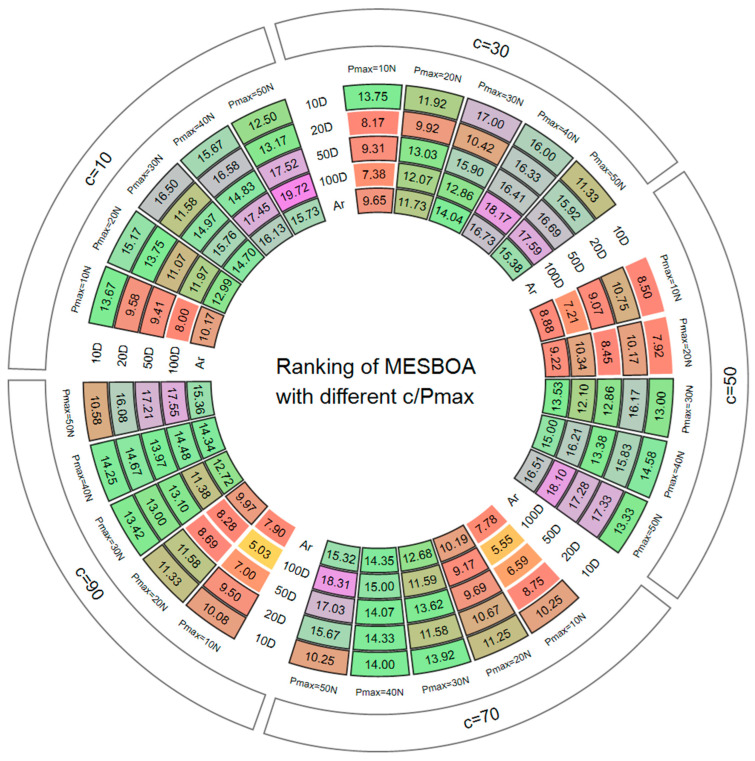
The Friedman ranking of MESBOA with different c/Pmax.

The EGS exerts its strength only when enough historical experience is stored and the correct exploration–exploitation bias is chosen; too few data fail to reveal the evolutionary trend, whereas too many may mislead and slow the search. Bcause different algorithms struggle to balance exploration and exploitation, we need to decide which behavior is currently required. Hence the best values of Pmax (history capacity) and c (bias control) are investigated. A grid search is again employed: Pmax is swept from 10 to 90 in steps of 20 and c from 10 to 50 in steps of 10. Figure 5 reports the Friedman ranks of MESBOA with different parameter pairs across all tested dimensions, where a lower average rank (Ar) indicates better overall performance. In Figure 5, the boxed numbers indicate the Friedman ranks obtained under each parameter configuration; the 10-D and 20-D results refer to the CEC 2022 suite, whereas the 50-D and 100-D results correspond to the CEC 2017 suite. Taking c = 10 as an example, the cell aggregates the ranks (and their average) across the five Pmax settings for every dimensionality: specifically, when c = 10 and Pmax = 10 N, the algorithm achieves a rank of 13.67 on the 10-D CEC 2022 functions.

At any fixed value of c, increasing Pmax consistently worsens the rank, implying that excessive historical information obscures rather than reveals the population’s evolutionary trend, and thereby misguides the search. Conversely, for a fixed Pmax, a larger c yields better ranks, showing that the original SBOA is exploration-deficient and benefits from stronger exploration bias introduced by EGS. The best compromise is obtained with c = 70 and Pmax = 10 N; this combination achieves the lowest Ar on both low- and high-dimensional functions, and is therefore adopted in all subsequent experiments.

### 4.4. Strategy Effectiveness Analysis

In this section, we examine the individual contributions of the proposed enhancement strategies to the performance gains observed in MESBOA. Two reduced variants, each incorporating only one of the two mechanisms, are evaluated—MSBOA, obtained by removing the EGS module from MESBOA, and ESBOA, derived by excluding the MMS component. The experimental results for MESBOA, SBOA, MSBOA and ESBOA on both test suites are compiled in Table A1, Table A2, Table A3 and Table A4 of Appendix A, and Friedman together with Wilcoxon rank-sum tests are employed to analyze the outcomes.

Table 2 summarizes the Friedman results for MESBOA and its two derived variants; the obtained *p*-value < 0.05 confirms significant differences among the four configurations. MESBOA, equipped with both enhancement modules, ranks first under all four dimensional settings, whereas the original SBOA always places last. MSBOA consistently outperforms ESBOA, indicating that the MMS component contributes more to the overall improvement than the EGS component, yet each single strategy still yields a clear gain over the baseline SBOA. Table 3 quantifies the win/tie/loss counts of MESBOA and its partial variants against SBOA: all three improved versions achieve significant superiority on more than half of the functions, again underlining the effectiveness of the proposed modifications. The number of wins for MSBOA exceeds that for ESBOA, further evidencing that MMS provides a larger performance boost than EGS. Overall, both proposed enhancement strategies are statistically validated as distinctly beneficial.

### 4.5. Low-Dimensional Function Experiments

**Figure 6:**
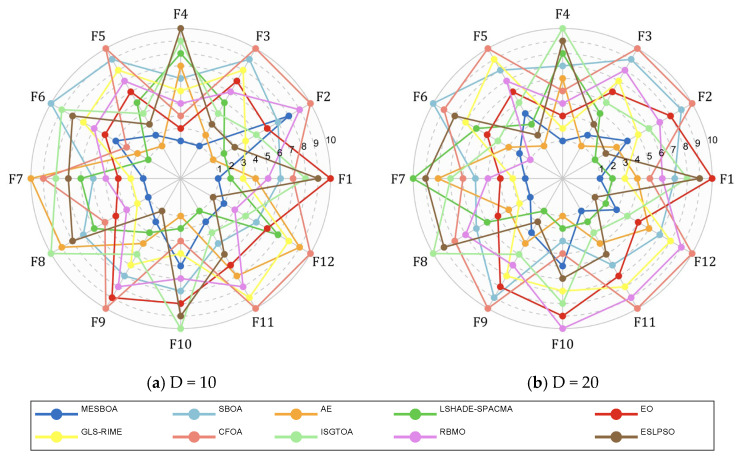
Radar diagram of MESBOA and comparison algorithms on CEC2022.

To comprehensively evaluate the performance of the proposed MESBOA on low-dimensional problems, the 12 functions of the CEC2022 test suite are adopted as the benchmark. Figure 6 shows the average rankings of MESBOA, AE, LSHADE-SPACMA, EO, GLS-RIME, CFOA, ISGTOA, RBMO and ESLPSO when solving the 10-D/20-D functions. The complete data of the best, mean and standard deviation values are provided in Table A1 and Table A2 of Appendix A. In the radar chart of rankings, each algorithm connects its ranks on the different functions into one surface; the smaller the enclosed surface, the better the overall performance. As can be seen, the surface produced by the proposed MESBOA is the smallest, demonstrating its superior overall performance. The surfaces of ESLPSO and ISGTOA are similar in size but exhibit poorer smoothness, indicating relatively weak stability and search efficiency. Although SBOA shows small fluctuations, its area is still large, confirming its poor overall performance.

**Figure 7:**
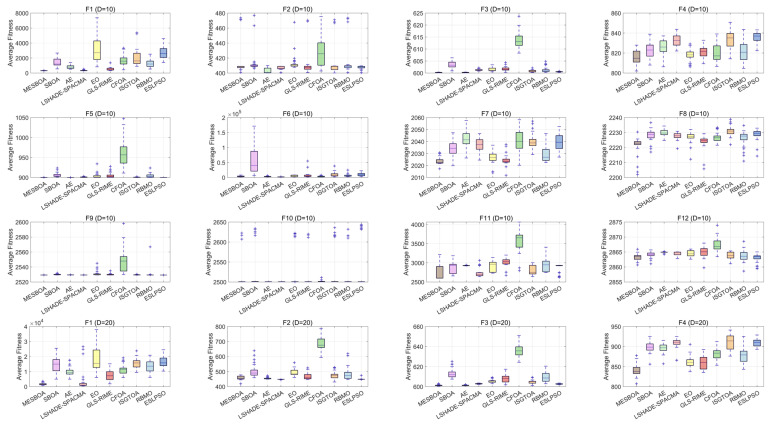

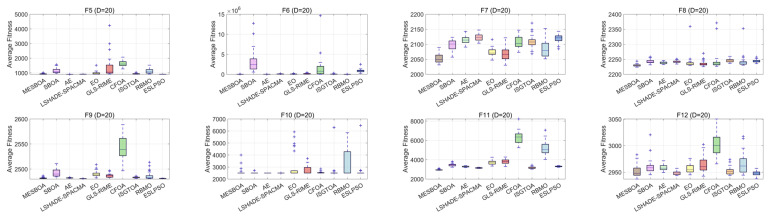
Box plots of MESBOA and comparison algorithms on CEC2022.

Figure 7 presents boxplots of MESBOA, AE, LSHADE-SPACMA, EO, GLS-RIME, CFOA, ISGTOA, RBMO and ESLPSO on the CEC2022 functions to assess robustness. It is evident that MESBOA exhibits the smallest box ranges on the majority of problems—specifically F1, F3–F4 and F7–F8 for 10-D, and F1, F4, F7–F8 and F11 for 20-D, ten functions in total—while also showing fewer outliers (“+”), indicating high stability. The plots further reveal that MESBOA consistently attains the lowest median on almost half of the functions, underscoring its superior accuracy. Overall, the narrow and low-lying boxes demonstrate that MESBOA delivers stable distributions and strong robustness across the test suite.

**Figure 8:**
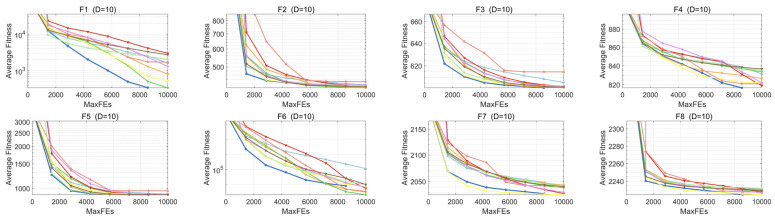

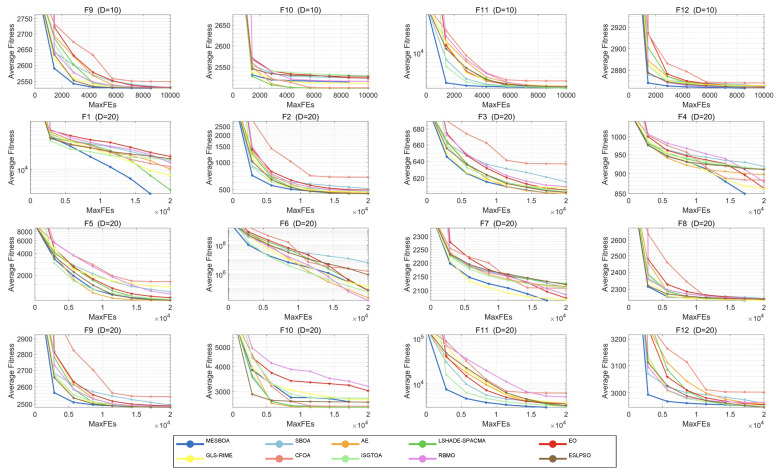
Convergence curves of MESBOA and comparison algorithms on CEC2022.

Based on the convergence curves collected on the CEC2022 test set, we further examined MESBOA’s convergence behavior when tackling low-dimensional tasks. As Figure 8 shows, MESBOA delivers excellent convergence. For the unimodal F1, all algorithms continue to converge, yet MESBOA exhibits the fastest speed and the highest accuracy, whereas the original SBOA descends much more slowly and attains a poorer final value. This superior performance on unimodal landscapes is attributed to MMS, which reconstructs the search framework so that dominant birds keep exploiting promising regions, and to EGS, which intensifies exploitation exactly when the population needs it; the synergy of the two mechanisms markedly strengthens MESBOA’s local search capability. On multimodal functions F2–F5, MESBOA is not always the most accurate on F2–F3 and F5, yet it converges faster and consistently yields higher-quality solutions than the basic SBOA. On F4 its convergence is slower, but the algorithm keeps escaping local optima and continues to discover better points. Compared with the original SBOA, MESBOA maintains a steady convergence curve, rarely stalls, and even accelerates in the later search phase. These improvements are credited to the supplementary role of the inferior group defined by MMS; by enlarging the search scope it helps the swarm jump out of local traps. In addition, EGS preserves population diversity through multiple guiding points, which further protects MESBOA from premature convergence. On the more challenging F6–F12, MESBOA attains the highest convergence accuracy on 10-D F7–F8, 20-D F7–F8 and F11. For the remaining functions except F10, it may not deliver the best final value, yet it always exhibits a rapid convergence rate; on F10 its speed and accuracy are not the top but they still surpass those of the basic SBOA. These results confirm that MESBOA possesses a well-balanced exploration–exploitation capability, owing to the fact that EGS dynamically detects the swarm’s current demand and drives each dimension to search accordingly. Overall, MESBOA demonstrates the best convergence behavior among all contenders on low-dimensional optimization problems.

In addition to convergence and robustness analyses, several statistical tests are employed to examine the differences between MESBOA and the compared algorithms. Table 4 summarizes the Wilcoxon rank-sum test results between MESBOA and AE, LSHADE-SPACMA, EO, GLS-RIME, CFOA, ISGTOA, RBMO, and ESLPSO, where the symbols “+/=/−” indicate that the proposed MESBOA is superior, similar, or inferior to the compared algorithm, respectively. The Wilcoxon rank-sum test is a non-parametric pairwise comparison method that checks whether two algorithms exhibit significant differences across different functions. It should be noted that all subsequent statistical tests are conducted at a significance level of 0.05. Table 4 shows that, against every competitor, MESBOA obtains more “+” than “−”; for most algorithms the count of “+” even exceeds the sum of “−” and “=”, evidencing a clear superiority. Versus the basic algorithms the advantage is larger in 20 D than in 10 D, whereas versus the enhanced variants the advantage is larger in 10 D than in 20 D. These patterns not only confirm the overall competitiveness of MESBOA but also re-assert the NFL statement that no single algorithm is best for all problems.

Beyond pairwise comparisons, an overall analysis is conducted using the Friedman test, and the results are reported in Table 5. The obtained *p*-values confirm a significant global performance difference between MESBOA and all contenders. Specifically, MESBOA ranks first on both 10-D and 20-D problems, achieving mean ranks of 2.500 and 2.333, respectively, followed immediately by LSHADE-SPACMA and AE, while the original SBOA places second-to-last, outperforming only CFOA. Notably, although MESBOA’s superiority margin is smaller in 20-D than in 10-D, its absolute rank is actually better at 20-D because the rankings of nearly all improved algorithms rise with dimension, while those of the baseline methods fall; consequently, MESBOA’s relative advantage over the other enhanced variants does not increase even though its absolute rank improves. Nevertheless, MESBOA unquestionably delivers the best overall performance among all algorithms examined.

**Figure 9:**
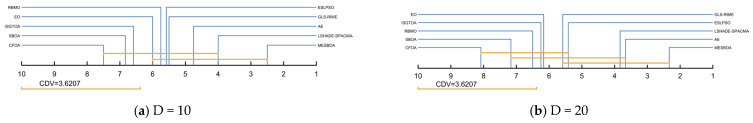
Nemenyi post-hoc test results of MESBOA and comparison algorithms on CEC2022.

The Friedman test only indicates whether significant differences exist among all algorithms; it does not quantify the magnitude of the gap between MESBOA and any specific competitor. Therefore, the Nemenyi post-hoc test is applied to obtain a finer-grained analysis. Based on the Friedman rankings, Nemenyi’s procedure computes a critical difference value (CDV) with which algorithm pairs can be judged equivalent; if the difference between the mean ranks of MESBOA and another algorithm is smaller than CDV, the two methods are deemed statistically indistinguishable. CDV is calculated with Equation (18).

(18) $$CDV=q_{a}\times \sqrt{\frac{K\left(K+1\right)}{6M}}$$

where M represents the number of algorithms and K represents the number of functions tested. $q_{a}$ is obtained from the table and equals 3.1640 in this paper. Figure 9 illustrates the Nemenyi post-hoc test results for MESBOA and the competing algorithms. It can be observed that MESBOA exhibits significant differences with CFOA, SBOA, and ISGTOA on 10-D functions, while no significant differences are found with the remaining algorithms. On 20-D functions, MESBOA shows significant differences with CFOA, SBOA, RBMO, ISGTOA, and EO, but not with the other algorithms. Therefore, it can be concluded that MESBOA is a competitive algorithm on the CEC2022 test suite.

### 4.6. High-Dimensional Function Experiments

**Figure 10:**
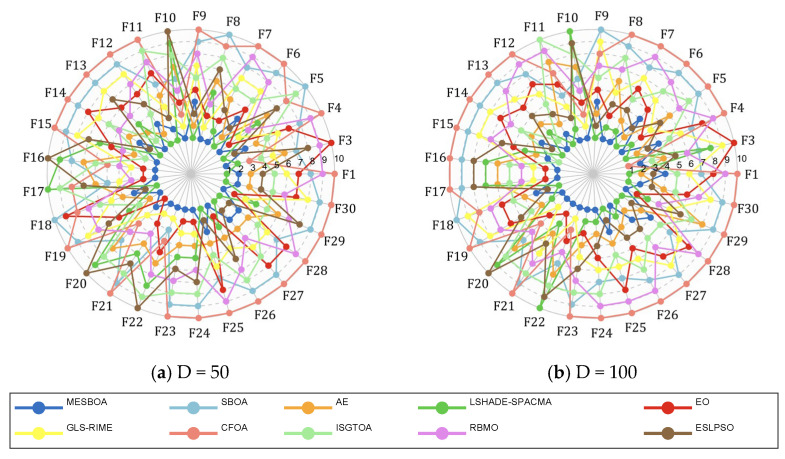
Radar diagram of MESBOA and comparison algorithms on CEC2017.

Although Section 4.5 has verified the effectiveness of MESBOA on low-dimensional tasks, modern applications increasingly involve high-dimensional and complex landscapes; hence the performance of MESBOA under such conditions must be assessed. The 50-D and 100-D instances of the CEC2017 test set are therefore employed. Figure 10 gives a first overview of the comparative behavior through a ranking radar chart, where the area enclosed by each algorithm indicates its overall standing on the CEC2017 benchmark. MESBOA never drops out of the top four ranks on any function, demonstrating consistently superior and stable performance, while SBOA and CFOA remain firmly in the bottom two positions across the entire benchmark.

**Figure 11:**
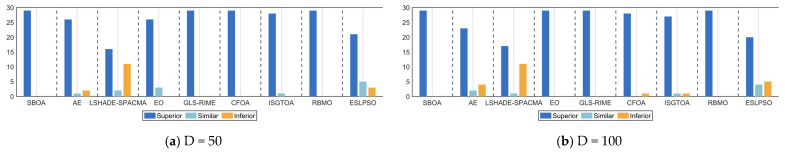
The number of “+/=/−” obtained by MESBOA and comparison algorithms on CEC2017.

Table 6 summarizes the Wilcoxon rank-sum results between MESBOA and the competitors on high-dimensional functions, with a visual depiction in Figure 11. MESBOA registers significant superiority on at least 16 functions against every rival. Specifically, the counts of superior/(inferior) functions are 58(0) vs. SBOA, 49(6) vs. AE, 33(22) vs. LSHADE-SPACMA, 55(0) vs. EO, 88(0) vs. GLS-RIME, 57(1) vs. CFOA, 55(1) vs. ISGTOA, 58(0) vs. RBMO, and 41(8) vs. ESLPSO. Overall, MESBOA exhibits clear advantages on the majority of 50-D and 100-D functions.

**Figure 12:**
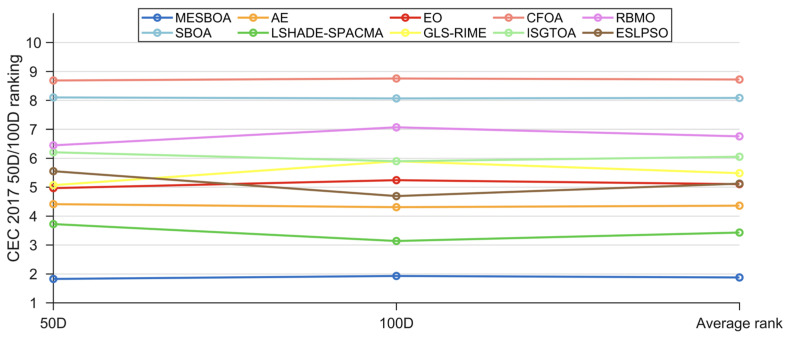
The Friedman ranking of MESBOA and comparison algorithms on CEC2017.

**Figure 13:**
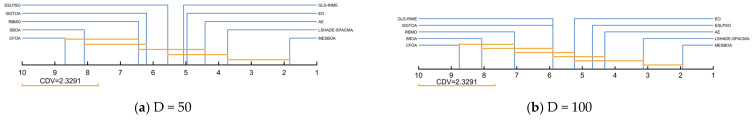
Nemenyi post-hoc test results of MESBOA and comparison algorithms on CEC2017.

The Friedman test results for MESBOA and the competing algorithms on the 50-D/100-D functions of the CEC2017 suite are reported in Table 7 and visualized in Figure 12; the *p*-values confirm a significant overall difference, which is further quantified by the Nemenyi post-hoc analysis shown in Figure 13. MESBOA ranks first on both dimensionalities with mean ranks of 1.828 (50-D) and 1.931 (100-D), whereas the original SBOA places second-to-last at 8.103 and 8.069, respectively, and LSHADE-SPACMA and AE occupy the second and third positions. The Nemenyi post-hoc test reveals that, on both 50-D and 100-D CEC2017 functions, MESBOA is not statistically distinguishable from LSHADE-SPACMA, yet it exhibits significant differences from all other contenders. This outcome contrasts with the CEC2022 low-dimensional results and indicates that the proposed MESBOA possesses a stronger edge when tackling high-dimensional problems. Moreover, being compared against the advanced differential evolution variant LSHADE-SPACMA further highlights the superiority of the proposed approach.

## 5. Engineering Optimization Problems Results and Analysis

To verify the effectiveness and robustness of MESBOA in solving real-world engineering optimization problems, three typical engineering design problems with multiple inequality constraints are selected. When constraints are violated, the penalty function method is adopted: any infeasible solution receives a large penalty value and is thus eliminated during iterations. To guarantee fairness and reproducibility, all tests are conducted under identical settings and each problem is run 30 independent times.

### 5.1. Pressure Vessel Design Problem

**Figure 14:**
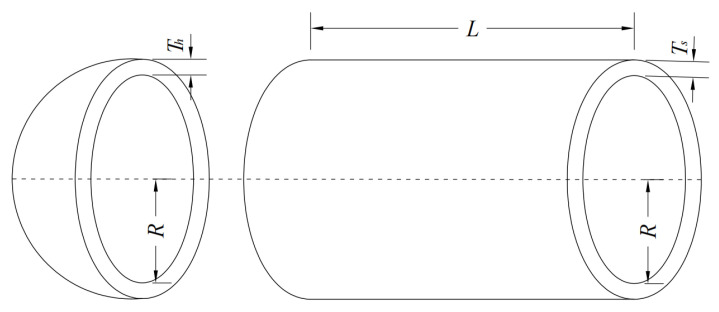
Schematic of pressure vessel.

The pressure vessel design optimization problem aims to minimize the structural weight of the material employed. It involves four design variables: shell thickness ($T_{s}$), head thickness ($T_{h}$), inner radius (R) and cylindrical shell length (L), collectively denoted as $\left[y_{1},y_{2},y_{3},y_{4}\right]$. The corresponding structural sketch is shown in Figure 14. Mathematically, it is shown as below.

Consider variable

(19) $$Y=[y_{1},y_{2},y_{3},y_{4}]=[T_{s},T_{h},R,L]$$

Minimize

(20) $$f(y)=0.6224y_{1}y_{3}y_{4}+1.7781y_{2}y_{3}^{2}+3.1661y_{1}^{2}y_{4}+19.84y_{1}^{2}y_{3}$$

Subject this to

(21) $$\begin{array}{c} g_{1}(Y)=-y_{1}+0.0193y_{2}\leq 0\\ g_{2}(Y)=-y_{2}+0.00954y_{3}\leq 0\\ g_{3}(Y)=-\pi y_{2}y_{3}^{2}y_{4}-\frac{4}{3}\pi y_{3}^{3}+1,296,000\leq 0\\ g_{4}(Y)=y_{4}-240\leq 0 \end{array}$$

The proposed MESBOA algorithm achieves the minimum weight of 5734.9131570 in the pressure vessel design problem, with the corresponding design variables shown in Table 8. This result is the best among all competitors, and the standard deviation over 30 runs is significantly lower than that of the other methods.

### 5.2. Welded Beam Design Problem

**Figure 15:**
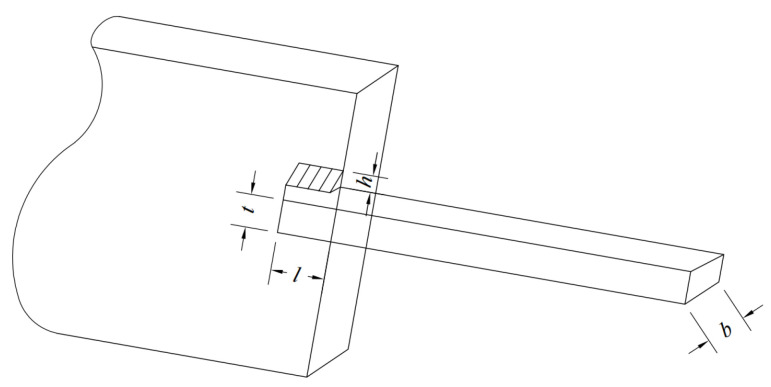
Schematic of welded beam design.

The welded beam design problem is a constrained optimization task whose objective is to minimize the weight of the beam. The design variables are four geometric parameters: weld thickness h, beam length l, beam thickness t and weld width b. The objective function computes the beam weight from these geometric quantities, while a set of constraints guarantees structural safety and feasibility. The corresponding structural sketch is shown in Figure 15. The mathematical model of the welded beam design problem is formulated as follows.

Consider variable

(22) $$Y=[y_{1,}y_{2},y_{3},y_{4}]=[h,l,t,b]$$

Minimize

(23) $$f(y)=1.10471y_{1}^{2}y_{2}+0.04811y_{3}y_{4}(14+y_{2})$$

Subject this to

(24) $$\begin{array}{l} g_{1}(Y)=\tau (y)-\tau_{\max}\leq 0\\ g_{2}(Y)=\sigma (y)-\sigma_{\max}\leq 0\\ g_{3}(Y)=\delta (y)-\delta_{\max}\leq 0\\ g_{4}(Y)=y_{1}-y_{4}\leq 0\\ g_{5}(Y)=P-P_{c}(x)\leq 0\\ g_{6}(Y)=0.125-y_{1}\leq 0\\ g_{7}(Y)=1.10471y_{1}^{2}+0.04811y_{3}y_{4}(14+y_{2})-5\leq 0 \end{array}$$

As shown in Table 9, the proposed MESBOA demonstrates superior performance on this problem, achieving the best fitness value of 1.724528 with the solution comprising $\left[y_{1,}y_{2},y_{3},y_{4}\right]$ = [0.187155, 3.470487, 9.036624, 0.205730].

### 5.3. Three-Bar Truss Design Problem

**Figure 16:**
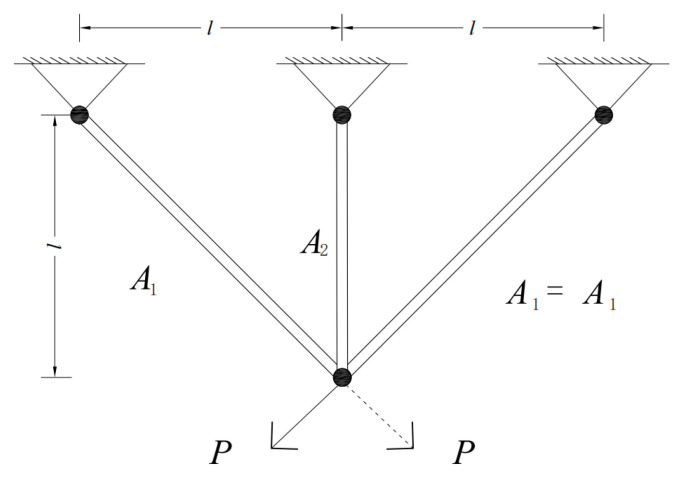
Schematic of three-bar truss design.

The three-bar truss design aims to produce a truss with the lowest possible weight while satisfying prescribed limits on deflection, buckling, and stress. This optimization problem involves two design variables, as shown in Figure 16, and its mathematical formulation is given below.

Consider variable

(25) $$Y=[y_{1},y_{2}]=[A_{1},A_{2}]$$

Minimize

(26) $$f(Y)=l\times \left(2\sqrt{2}y_{1}+y_{2}\right)$$

Subject this to

(27) $$\begin{array}{l} g_{1}(Y)\frac{\sqrt{2}y_{1}+y_{2}}{\sqrt{2}y_{1}^{2}+2y_{1}y_{2}}P-\sigma ⩽0\\ g_{2}(Y)=\frac{y_{2}}{\sqrt{2}y_{1}^{2}+2y_{1}y_{2}}P-\sigma ⩽0\\ g_{3}(Y)=\frac{1}{y_{1}+\sqrt{2}y_{2}}P-\sigma ⩽0 \end{array}$$

As shown in Table 10, although the differences between MESBOA and the competing algorithms are small, MESBOA still delivers the best solution, with a corresponding fitness value of 263.8523464.

## 6. Conclusions

This paper presents MESBOA, an SBOA variant that addresses the original algorithm’s imbalance between exploitation and exploration and its tendency to become trapped in local optima. A multi-population management strategy (MMS) restructures the SBOA search framework to coordinate exploitation and exploration, while an Experience-Guided Strategy (EGS) dynamically captures the evolutionary trend of the population and regulates exploration/exploitation on demand. Leveraging the guiding role of dominant swarms, MESBOA enhances global exploration, maintains diversity and accelerates convergence. Simulation experiments on the CEC2017 and CEC2022 test suites demonstrate that the proposed MESBOA outperforms eight state-of-the-art algorithms in terms of optimization capability and convergence performance. In addition, experiments on engineering constrained optimization problems show its strong advantages in real-world optimization tasks.

Admittedly, MESBOA still has limitations that warrant further attention. First, the newly introduced parameters of EGS and MMS must be manually tuned, restricting its broader applicability. Second, low-dimensional benchmarks reveal only marginal advantages, indicating that its performance on small-scale problems needs improvement. Finally, although EGS is invoked sparingly, the embedded covariance matrix computations are time-consuming, hindering deployment in real-time optimization. Future work will therefore focus on three directions: (1) adopting reinforcement learning or other adaptive parameter control techniques to eliminate manual tuning and widen the application domain; (2) embedding search operators specifically designed for low-dimensional landscapes and equipping the algorithm with a landscape-detection module to select operators dynamically; and (3) accelerating the covariance update via parallel architectures or matrix-skeletonization so that the method can tackle dynamic optimization tasks with tight timing constraints.

## Figures and Tables

**Table 1:** Parameter setting of each algorithm.

Algorithm	Parameters
MESBOA	N=15D,η=1.5,a=0.4,b=0.7,c=70,Pmax=10D
SBOA	N=30,η=1.5
AE	N=30,a=4
LSHADE-SPACMA	$N=18D,N_{\min}=4,P=0.11,\left|A\right|=1.4,h=5$
EO	$N=100,a_{1}=2,a_{2}=1,GP=0.5$
GLS-RIME	N=30,W=5,A=60,Cmax=3000
CFOA	N=30
ISGTOA	N=50,b+c=1
RBMO	N=30,E=0.5
ESLPSO	N=30,uCR=0.5,uF=0.5,c=0.1

**Table 2:** Friedman test results of MESBOA and its derived algorithms.

Test Suite	Dimension	SBOA	MSBOA	ESBOA	MESBOA	*p*-Value
CEC2022	10	3.750	2.250	2.500	1.500	2.87 × 10^−4^
	20	3.750	2.417	2.500	1.333	1.00 × 10^−4^
CEC2017	50	3.966	2.000	2.931	1.103	5.31 × 10^−17^
	100	4.000	1.966	2.931	1.103	1.62 × 10^−17^
Average rank		3.866	2.158	2.716	1.260	N/A
Overall rank		4	2	3	1	N/A

**Table 3:** Wilcoxon rank sum test results of MESBOA and its derived algorithms.

vs. SBOA (+/=/−)	CEC2022 Test Suite		CEC2017 Test Suite	
	10 D	30 D	50 D	100 D
MSBOA	11/0/1	11/0/1	27/2/0	29/0/0
ESBOA	9/2/1	11/0/1	25/4/0	28/1/0
MESBOA	11/0/1	11/0/1	29/0/0	29/0/0

**Table 4:** Wilcoxon rank sum test results of MESBOA and comparison algorithms on CEC2022.

MESBOA vs. +/=/−		SBOA	AE	LSHADE-SPACMA	EO	GLS-RIME	CFOA	ISGTOA	RBMO	ESLPSO
CEC-2022 test suite	10D	11/0/1	7/3/2	8/3/1	9/2/1	9/3/0	10/1/1	11/1/0	8/4/0	8/3/1
	20D	11/0/1	8/3/1	6/2/4	12/0/0	11/1/0	11/0/1	10/2/0	11/1/0	7/2/3

**Table 5:** Friedman test results of MESBOA and comparison algorithms on CEC2022.

Test Suite	CEC-2022	MESBOA	SBOA	AE	LSHADE-SPACMA	EO	GLS-RIME	CFOA	ISGTOA	RBMO	ESLPSO	*p*-Value
Dimension	10	2.500	6.833	4.750	4.000	6.000	5.500	7.500	6.583	5.750	5.583	2.99 × 10^−3^
	20	2.333	7.167	3.667	3.833	6.167	5.583	8.083	6.250	6.500	5.417	5.42 × 10^0^
Average rank		2.417	7.000	4.208	3.917	6.083	5.542	7.792	6.417	6.125	5.500	N/A
Overall rank		1	9	3	2	6	4	10	8	7	5	N/A

**Table 6:** Wilcoxon rank sum test results of MESBOA and comparison algorithms on CEC2017.

MESBOA vs. +/=/−		SBOA	AE	LSHADE-SPACMA	EO	GLS-RIME	CFOA	ISGTOA	RBMO	ESLPSO
CEC-2017 test suite	50D	29/0/0	26/1/2	16/2/11	26/3/0	29/0/0	29/0/0	28/1/0	29/0/0	21/5/3
	100D	29/0/0	23/2/4	17/1/11	29/0/0	29/0/0	28/0/1	27/1/1	29/0/0	20/4/5

**Table 7:** Friedman test results of MESBOA and comparison algorithms on CEC2017.

Test Suite	CEC-2017	MESBOA	SBOA	AE	LSHADE-SPACMA	EO	GLS-RIME	CFOA	ISGTOA	RBMO	ESLPSO	*p*-Value
Dimension	50	1.828	8.103	4.414	3.724	4.966	5.069	8.690	6.207	6.448	5.552	9.07 × 10^−21^
	100	1.931	8.069	4.310	3.138	5.241	5.897	8.759	5.897	7.069	4.690	9.07 × 10^−21^
Average rank		1.879	8.086	4.362	3.431	5.103	5.483	8.724	6.052	6.759	5.121	N/A
Overall rank		1	9	3	2	4	6	10	7	8	5	N/A

**Table 8:** Results of MESBOA and competitors on the pressure vessel design problem.

Algorithm	Optimal Values				Min	Avg	Std
	Ts	Th	R	L			
MESBOA	0.7424336	0.3701961	40.3196187	200	5734.9131570	5734.9132223	0.0001859
SBOA	1.0105148	0.4865822	53.6884007	74.2060890	6458.6563412	6658.6898335	489.7784916
AE	0.7678084	0.3867046	41.5987812	186.5353308	5865.9261129	5870.1240243	12.8355450
LSHADE-SPACMA	0.7424356	0.3701942	40.3196187	200	5734.9131634	5750.2190817	47.0773456
EO	0.7216561	0.3554438	40.4484786	200	5856.0091856	6012.4315885	97.0115631
GLS-RIME	0.8407224	0.4058078	44.6692450	147.3116683	5926.2125862	6591.0809350	427.1301470
CFOA	1.0105148	0.4865822	53.6884007	74.2060890	6458.6563412	7510.0983382	731.9024371
ISGTOA	0.7362395	0.4161056	40.6854150	194.9697617	5871.7764090	5874.9531943	40.1885280
RBMO	0.7784057	0.3854974	40.3230745	200	5890.0709671	6022.9784136	152.7715233
ESLPSO	0.7781687	0.3846498	40.3196187	200	5885.3357162	5870.1239766	475.4031033

**Table 9:** Results of MESBOA and competitors on the welded beam design problem.

Algorithm	Optimal Values				Min	Avg	Std
	h	l	t	b			
MESBOA	0.187155	3.470487	9.036624	0.205730	1.724528	1.7245791	0.0000123
SBOA	0.2057301	3.4704767	9.0366592	0.2057300	1.7248600	1.9528762	0.1802129
AE	0.203687	3.470489	9.036624	0.205729	1.724718	1.7248689	0.0000364
LSHADE-SPACMA	0.187156	3.744548	9.173438	0.205057	1.724536	1.7253144	0.0005242
EO	0.205700	3.657587	9.176331	0.205111	1.733487	1.7632446	0.0296859
GLS-RIME	0.205701	3.479005	9.036874	0.205732	1.733462	1.7540066	0.0522504
CFOA	0.2042892	3.5006486	9.2297572	0.2084563	1.7813186	1.9275891	0.3089531
ISGTOA	0.2057280	3.4705097	9.0366974	0.2057325	1.7248870	1.8457670	0.0321628
RBMO	0.204368	3.856979	3.856979	0.212148	1.729843	1.7722041	0.0390293
ESLPSO	0.205729	3.512662	8.997062	0.207548	1.724852	1.7270023	0.0369626

**Table 10:** Results of MESBOA and competitors on the three-bar truss design problem.

Algorithm	Optimal Values		Min	Avg	Std
	A_1_	A_2_			
MESBOA	0.7884152	0.4081138	263.8523464	263.8523464	1.1563 × 10^−13^
SBOA	0.7888521	0.4076515	263.8915722	264.6893199	1.00855621
AE	0.7884593	0.4079891	263.8523479	263.8523481	6.13525 × 10^−6^
LSHADE-SPACMA	0.7882057	0.4087165	263.8523850	263.8523473	0.00274232
EO	0.7885426	0.4085384	263.8915031	263.8914911	4.39972 × 10^−8^
GLS-RIME	0.7887717	0.4071060	263.8524398	263.8915398	0.03907688
CFOA	0.7885974	0.4083795	263.8914940	263.8964474	0.01162702
ISGTOA	0.7884593	0.4079891	263.8523479	263.8988462	0.01332436
RBMO	0.7883327	0.4091367	263.8915894	264.0411967	0.23354843
ESLPSO	0.7884444	0.4080303	263.8523471	263.8914911	0.02359312

## Data Availability

All data supporting the findings of this study are available within the paper.

## Appendix A

**Table A1:** The statistical results of MESBOA and its derived algorithms on the CEC 2022 in dimensions of 10.

No.	Index	SBOA	MSBOA	ESBOA	MESBOA
F1	Min	5.5352E+02	3.1561E+02	3.2611E+02	3.0008E+02
	Avg	1.4071E+03	4.1585E+02	3.8985E+02	3.1454E+02
	Std	6.0831E+02	1.2941E+02	3.7755E+01	1.3722E+01
F2	Best	4.0601E+02	4.0035E+02	4.0024E+02	4.0086E+02
	Avg	4.1381E+02	4.1732E+02	4.0923E+02	4.1401E+02
	Std	1.5470E+01	2.5167E+01	1.2085E+01	1.9802E+01
F3	Best	6.0092E+02	6.0005E+02	6.0023E+02	6.0009E+02
	Avg	6.0350E+02	6.0067E+02	6.0078E+02	6.0021E+02
	Std	1.3381E+00	1.1736E+00	4.0234E−01	7.1382E−02
F4	Best	8.0797E+02	8.0703E+02	8.0748E+02	8.0200E+02
	Avg	8.2306E+02	8.1874E+02	8.1413E+02	8.1592E+02
	Std	7.5038E+00	8.2939E+00	5.0872E+00	6.6586E+00
F5	Best	9.0120E+02	9.0016E+02	9.0002E+02	9.0000E+02
	Avg	9.0615E+02	9.0311E+02	9.0027E+02	9.0009E+02
	Std	5.0025E+00	4.8743E+00	1.7386E−01	1.0028E−01
F6	Best	6.5012E+03	1.9935E+03	2.2026E+03	1.9415E+03
	Avg	5.3346E+04	5.2305E+03	4.7676E+03	4.0973E+03
	Std	4.1672E+04	2.0695E+03	1.8256E+03	1.9009E+03
F7	Best	2.0198E+03	2.0212E+03	2.0221E+03	2.0173E+03
	Avg	2.0346E+03	2.0231E+03	2.0280E+03	2.0237E+03
	Std	6.9307E+00	2.1432E+00	3.5513E+00	2.9332E+00
F8	Best	2.2170E+03	2.2064E+03	2.2156E+03	2.2020E+03
	Avg	2.2280E+03	2.2233E+03	2.2263E+03	2.2213E+03
	Std	3.9364E+00	3.5381E+00	3.7912E+00	6.3528E+00
F9	Best	2.5294E+03	2.5293E+03	2.5293E+03	2.5293E+03
	Avg	2.5300E+03	2.5293E+03	2.5294E+03	2.5293E+03
	Std	5.5915E−01	7.4202E−02	1.5615E−01	2.8182E−02
F10	Best	2.5004E+03	2.5003E+03	2.5003E+03	2.5003E+03
	Avg	2.5217E+03	2.5083E+03	2.5235E+03	2.5155E+03
	Std	4.7554E+01	2.9716E+01	4.6946E+01	3.9134E+01
F11	Best	2.6578E+03	2.6005E+03	2.6115E+03	2.6004E+03
	Avg	2.8522E+03	2.7853E+03	2.7897E+03	2.7765E+03
	Std	1.4014E+02	1.6902E+02	1.5570E+02	1.7604E+02
F12	Best	2.8611E+03	2.8596E+03	2.8596E+03	2.8607E+03
	Avg	2.8641E+03	2.8629E+03	2.8636E+03	2.8632E+03
	Std	9.7370E−01	1.2616E+00	1.4768E+00	1.1067E+00

**Table A2:** The statistical results of MESBOA and its derived algorithms on the CEC 2022 in dimensions of 20.

No.	Index	SBOA	MSBOA	ESBOA	MESBOA
F1	Min	5.0359E+03	1.0833E+03	1.7139E+03	8.3858E+02
	Avg	1.4221E+04	5.1301E+03	3.3450E+03	1.6891E+03
	Std	5.1642E+03	2.9662E+03	9.0029E+02	6.9714E+02
F2	Best	4.5978E+02	4.4510E+02	4.4085E+02	4.1915E+02
	Avg	5.0292E+02	4.6441E+02	4.5981E+02	4.5938E+02
	Std	4.3153E+01	1.2992E+01	1.4308E+01	1.3063E+01
F3	Best	6.0755E+02	6.0062E+02	6.0216E+02	6.0053E+02
	Avg	6.1309E+02	6.0293E+02	6.0474E+02	6.0128E+02
	Std	4.4226E+00	1.5536E+00	1.5033E+00	5.9607E−01
F4	Best	8.5622E+02	8.2256E+02	8.4880E+02	8.0729E+02
	Avg	8.9906E+02	8.5261E+02	8.7853E+02	8.4025E+02
	Std	1.3812E+01	1.7090E+01	1.4215E+01	1.4961E+01
F5	Best	9.7435E+02	9.1135E+02	9.0822E+02	9.0177E+02
	Avg	1.1351E+03	1.0540E+03	9.4429E+02	9.2697E+02
	Std	1.5742E+02	1.1423E+02	4.0574E+01	2.7455E+01
F6	Best	5.6861E+05	2.1926E+03	1.0887E+05	2.4415E+03
	Avg	3.2073E+06	1.8696E+04	4.4962E+05	1.6519E+04
	Std	2.7864E+06	1.3436E+04	2.3261E+05	1.4728E+04
F7	Best	2.0573E+03	2.0273E+03	2.0483E+03	2.0314E+03
	Avg	2.0973E+03	2.0630E+03	2.0829E+03	2.0525E+03
	Std	1.7843E+01	2.3158E+01	1.9506E+01	1.5269E+01
F8	Best	2.2315E+03	2.2239E+03	2.2297E+03	2.2239E+03
	Avg	2.2426E+03	2.2308E+03	2.2385E+03	2.2297E+03
	Std	6.2336E+00	5.4370E+00	3.9057E+00	4.0834E+00
F9	Best	2.4844E+03	2.4808E+03	2.4816E+03	2.4809E+03
	Avg	2.4926E+03	2.4820E+03	2.4836E+03	2.4820E+03
	Std	7.4879E+00	1.4145E+00	1.5079E+00	1.2667E+00
F10	Best	2.5006E+03	2.5004E+03	2.5005E+03	2.5005E+03
	Avg	2.5296E+03	2.6710E+03	2.5468E+03	2.6017E+03
	Std	7.3987E+01	4.6806E+02	8.4983E+01	3.1332E+02
F11	Best	3.2248E+03	2.9032E+03	3.0228E+03	2.9085E+03
	Avg	3.4671E+03	3.0928E+03	3.0923E+03	2.9622E+03
	Std	1.4417E+02	1.5658E+02	4.9011E+01	5.4120E+01
F12	Best	2.9452E+03	2.9365E+03	2.9406E+03	2.9370E+03
	Avg	2.9608E+03	2.9504E+03	2.9524E+03	2.9526E+03
	Std	1.4357E+01	1.1311E+01	7.4899E+00	1.2159E+01

**Table A3:** The statistical results of MESBOA and its derived algorithms on the CEC 2017 in dimensions of 50.

No.	Index	SBOA	MSBOA	ESBOA	MESBOA
F1	Min	5.3234E+09	7.6065E+07	1.3221E+09	1.4560E+07
	Avg	6.7391E+09	5.1368E+08	2.7944E+09	2.3648E+08
	Std	1.0966E+09	3.4326E+08	1.3177E+09	2.3129E+08
F3	Best	1.1419E+05	7.0729E+04	6.9889E+04	6.5945E+04
	Avg	1.5577E+05	1.0090E+05	1.0036E+05	8.8189E+04
	Std	2.4727E+04	1.5933E+04	2.3988E+04	1.4670E+04
F4	Best	9.2567E+02	5.5087E+02	7.3922E+02	5.5091E+02
	Avg	1.2961E+03	6.9143E+02	8.4063E+02	6.3903E+02
	Std	2.5555E+02	9.4532E+01	9.3296E+01	6.3674E+01
F5	Best	8.7112E+02	6.4803E+02	8.6313E+02	6.0263E+02
	Avg	9.4864E+02	7.0429E+02	8.9166E+02	6.5239E+02
	Std	4.5724E+01	4.9281E+01	3.4187E+01	3.5460E+01
F6	Best	6.3411E+02	6.1355E+02	6.1731E+02	6.0746E+02
	Avg	6.4201E+02	6.2025E+02	6.2652E+02	6.1328E+02
	Std	5.2349E+00	3.3361E+00	6.3280E+00	2.9741E+00
F7	Best	1.2417E+03	9.7991E+02	1.1605E+03	9.2451E+02
	Avg	1.3345E+03	1.0961E+03	1.2401E+03	9.8456E+02
	Std	6.5223E+01	6.9965E+01	4.5451E+01	4.6264E+01
F8	Best	1.2189E+03	9.6391E+02	1.1566E+03	9.4115E+02
	Avg	1.2652E+03	1.0069E+03	1.2087E+03	9.7088E+02
	Std	2.5458E+01	3.4370E+01	3.4889E+01	2.0418E+01
F9	Best	6.6165E+03	1.9385E+03	5.0748E+03	1.8049E+03
	Avg	1.5164E+04	4.5938E+03	8.9684E+03	2.5104E+03
	Std	7.0642E+03	1.8993E+03	2.0750E+03	5.8870E+02
F10	Best	1.1968E+04	5.9223E+03	1.1760E+04	6.8605E+03
	Avg	1.3420E+04	7.4356E+03	1.3253E+04	7.7645E+03
	Std	8.6255E+02	1.2173E+03	1.0574E+03	8.6262E+02
F11	Best	2.3350E+03	1.5130E+03	1.7148E+03	1.3359E+03
	Avg	2.9779E+03	1.5781E+03	1.8813E+03	1.4879E+03
	Std	5.3114E+02	7.4236E+01	1.0822E+02	1.3059E+02
F12	Best	6.4145E+08	2.6219E+07	6.3167E+07	1.3154E+07
	Avg	1.0885E+09	4.7882E+07	2.0069E+08	3.3967E+07
	Std	2.6347E+08	2.1145E+07	7.0311E+07	1.4797E+07
F13	Best	3.8512E+07	2.2719E+05	1.0132E+06	6.0886E+04
	Avg	1.0187E+08	8.3743E+05	6.2779E+06	2.1344E+05
	Std	5.7538E+07	6.5209E+05	7.5739E+06	2.2413E+05
F14	Best	2.2823E+05	8.5110E+04	4.0584E+04	1.6070E+04
	Avg	8.7263E+05	5.0388E+05	1.3693E+05	1.3413E+05
	Std	4.7572E+05	2.9648E+05	5.4611E+04	8.5056E+04
F15	Best	1.2095E+06	5.0314E+04	6.0063E+04	1.8875E+04
	Avg	5.9296E+06	2.2444E+05	2.6860E+05	4.8664E+04
	Std	4.8641E+06	2.4641E+05	2.5129E+05	2.1482E+04
F16	Best	3.5814E+03	2.4757E+03	2.9499E+03	2.3811E+03
	Avg	4.6814E+03	3.2775E+03	3.7877E+03	3.0954E+03
	Std	4.9232E+02	3.6609E+02	5.6897E+02	4.1030E+02
F17	Best	3.3792E+03	2.6216E+03	3.0215E+03	2.7166E+03
	Avg	3.7703E+03	2.9528E+03	3.4059E+03	2.9606E+03
	Std	2.5021E+02	2.6651E+02	3.1684E+02	1.9923E+02
F18	Best	3.2424E+06	8.1453E+05	8.1007E+05	1.5310E+05
	Avg	7.0514E+06	4.6193E+06	1.5656E+06	1.3970E+06
	Std	2.9777E+06	3.3259E+06	9.5504E+05	1.1457E+06
F19	Best	2.0696E+06	1.1930E+04	2.4517E+04	4.4091E+03
	Avg	4.0114E+06	8.6761E+04	2.7574E+05	2.5381E+04
	Std	2.5494E+06	7.4527E+04	3.3317E+05	1.9620E+04
F20	Best	3.2181E+03	2.6229E+03	2.6406E+03	2.4998E+03
	Avg	3.4996E+03	3.1417E+03	3.2717E+03	3.0325E+03
	Std	2.2515E+02	3.3210E+02	3.4044E+02	2.6853E+02
F21	Best	2.6926E+03	2.4298E+03	2.6541E+03	2.3792E+03
	Avg	2.7567E+03	2.5113E+03	2.6903E+03	2.4503E+03
	Std	4.3726E+01	5.7936E+01	2.5168E+01	3.5139E+01
F22	Best	3.2955E+03	8.6472E+03	1.3496E+04	7.8579E+03
	Avg	1.3487E+04	1.0069E+04	1.5109E+04	9.2700E+03
	Std	5.0832E+03	8.8145E+02	1.0637E+03	8.5983E+02
F23	Best	3.1260E+03	2.8921E+03	3.0990E+03	2.8408E+03
	Avg	3.1992E+03	2.9279E+03	3.1465E+03	2.8862E+03
	Std	4.4414E+01	3.0486E+01	3.6159E+01	3.3102E+01
F24	Best	3.3135E+03	3.0563E+03	3.2794E+03	3.0159E+03
	Avg	3.3580E+03	3.0961E+03	3.3223E+03	3.0519E+03
	Std	2.9522E+01	3.3142E+01	3.2887E+01	3.7292E+01
F25	Best	3.4077E+03	3.0950E+03	3.3067E+03	3.0471E+03
	Avg	3.5863E+03	3.2276E+03	3.4021E+03	3.1440E+03
	Std	1.2552E+02	8.9607E+01	7.8371E+01	6.2034E+01
F26	Best	8.1073E+03	5.5070E+03	7.4362E+03	4.7936E+03
	Avg	8.5829E+03	5.9440E+03	8.0917E+03	5.5319E+03
	Std	3.8883E+02	3.1599E+02	4.3166E+02	5.2125E+02
F27	Best	3.5133E+03	3.3665E+03	3.4427E+03	3.3330E+03
	Avg	3.6260E+03	3.4337E+03	3.5473E+03	3.4423E+03
	Std	1.1981E+02	6.3436E+01	6.0396E+01	6.2900E+01
F28	Best	3.8280E+03	3.4352E+03	3.5027E+03	3.3546E+03
	Avg	4.3097E+03	3.6739E+03	3.7625E+03	3.5227E+03
	Std	5.0512E+02	1.6768E+02	1.5463E+02	1.1589E+02
F29	Best	4.9456E+03	4.0244E+03	4.4871E+03	4.0078E+03
	Avg	5.6957E+03	4.5094E+03	5.0447E+03	4.3459E+03
	Std	6.5240E+02	3.2636E+02	3.5367E+02	2.6737E+02
F30	Best	4.7537E+07	7.2379E+06	1.1101E+07	4.2982E+06
	Avg	1.0429E+08	1.2192E+07	3.7042E+07	9.7981E+06
	Std	4.1100E+07	3.9284E+06	1.7023E+07	3.5740E+06

**Table A4:** The statistical results of MESBOA and its derived algorithms on the CEC 2017 in dimensions of 100.

No.	Index	SBOA	MSBOA	ESBOA	MESBOA
F1	Min	3.2980E+10	5.3269E+09	1.3690E+10	2.4318E+09
	Avg	4.2816E+10	1.3065E+10	2.4107E+10	5.3457E+09
	Std	5.8280E+09	5.6498E+09	5.2249E+09	2.6161E+09
F3	Best	3.3304E+05	2.7628E+05	2.7540E+05	2.4978E+05
	Avg	4.2239E+05	3.1456E+05	3.6654E+05	3.0379E+05
	Std	6.7452E+04	2.9059E+04	5.0303E+04	3.2432E+04
F4	Best	3.1605E+03	1.2686E+03	2.3453E+03	1.0421E+03
	Avg	4.9843E+03	1.7801E+03	2.8980E+03	1.2898E+03
	Std	1.2147E+03	3.1101E+02	3.9162E+02	1.8028E+02
F5	Best	1.5227E+03	9.0219E+02	1.3225E+03	8.5788E+02
	Avg	1.6376E+03	1.0772E+03	1.5029E+03	9.5578E+02
	Std	9.0749E+01	1.3021E+02	7.9265E+01	7.7632E+01
F6	Best	6.5335E+02	6.3164E+02	6.4098E+02	6.2682E+02
	Avg	6.6957E+02	6.4013E+02	6.5683E+02	6.3255E+02
	Std	8.6382E+00	4.6371E+00	7.9537E+00	3.5151E+00
F7	Best	2.2647E+03	1.7474E+03	1.9981E+03	1.3484E+03
	Avg	2.5539E+03	2.0686E+03	2.2654E+03	1.5934E+03
	Std	1.2597E+02	2.0011E+02	1.4981E+02	1.0417E+02
F8	Best	1.8248E+03	1.2802E+03	1.7357E+03	1.1585E+03
	Avg	1.9418E+03	1.4022E+03	1.8512E+03	1.2386E+03
	Std	7.6470E+01	1.0474E+02	6.7080E+01	4.7990E+01
F9	Best	4.5050E+04	1.4034E+04	2.3691E+04	8.3892E+03
	Avg	5.6746E+04	2.0006E+04	4.0845E+04	1.3159E+04
	Std	6.4116E+03	3.3595E+03	7.1214E+03	1.9466E+03
F10	Best	2.8668E+04	1.4079E+04	2.7618E+04	1.4870E+04
	Avg	3.0919E+04	1.6575E+04	2.9801E+04	1.7204E+04
	Std	1.0165E+03	1.6416E+03	1.0419E+03	1.1994E+03
F11	Best	3.1428E+04	6.1680E+03	1.5576E+04	3.9943E+03
	Avg	5.2745E+04	9.4430E+03	2.6609E+04	6.3384E+03
	Std	1.1647E+04	2.9734E+03	7.1705E+03	1.6039E+03
F12	Best	5.7051E+09	1.9982E+08	1.7899E+09	1.4451E+08
	Avg	8.9670E+09	5.9354E+08	2.6942E+09	4.3576E+08
	Std	1.7426E+09	5.4524E+08	6.4773E+08	2.5353E+08
F13	Best	2.6843E+08	1.1919E+05	1.1260E+07	1.3883E+05
	Avg	5.5023E+08	3.2892E+06	4.4907E+07	2.7317E+06
	Std	1.6736E+08	3.3607E+06	2.1978E+07	2.6033E+06
F14	Best	2.8949E+06	8.8110E+05	1.0196E+06	4.8300E+05
	Avg	9.2366E+06	3.9241E+06	2.7268E+06	1.4325E+06
	Std	4.8510E+06	2.4508E+06	1.1503E+06	5.8828E+05
F15	Best	3.3748E+07	1.0741E+05	8.8774E+05	3.8257E+04
	Avg	9.9888E+07	1.2207E+06	3.8167E+06	4.2459E+05
	Std	4.1774E+07	1.5002E+06	2.8864E+06	1.1653E+06
F16	Best	9.3623E+03	4.8568E+03	8.3591E+03	4.7741E+03
	Avg	1.0123E+04	6.0638E+03	9.2434E+03	5.6919E+03
	Std	5.8862E+02	6.6720E+02	4.9920E+02	6.0055E+02
F17	Best	6.9158E+03	4.5616E+03	4.9505E+03	3.7788E+03
	Avg	7.7357E+03	5.0284E+03	6.6494E+03	4.4434E+03
	Std	4.4106E+02	3.3799E+02	6.0683E+02	4.2674E+02
F18	Best	6.1337E+06	1.2209E+06	1.8866E+06	8.1386E+05
	Avg	1.2822E+07	4.6714E+06	4.3101E+06	2.8206E+06
	Std	5.2998E+06	1.7391E+06	2.0198E+06	1.4860E+06
F19	Best	6.0881E+07	7.7707E+05	2.3783E+06	2.5206E+05
	Avg	1.1988E+08	2.6216E+06	6.5689E+06	8.8844E+05
	Std	4.5012E+07	1.4133E+06	2.9252E+06	5.1209E+05
F20	Best	6.2813E+03	3.7534E+03	5.3885E+03	3.8666E+03
	Avg	6.8889E+03	4.8917E+03	6.3452E+03	4.7465E+03
	Std	3.9301E+02	6.4881E+02	5.9066E+02	5.2602E+02
F21	Best	3.3687E+03	2.7580E+03	3.2624E+03	2.6685E+03
	Avg	3.4851E+03	2.8750E+03	3.3326E+03	2.7679E+03
	Std	6.5771E+01	5.5995E+01	3.3046E+01	6.3311E+01
F22	Best	3.1014E+04	1.5486E+04	2.9022E+04	1.7083E+04
	Avg	3.3321E+04	1.9207E+04	3.2578E+04	1.9356E+04
	Std	1.0935E+03	1.8889E+03	1.2791E+03	1.7042E+03
F23	Best	3.8458E+03	3.2380E+03	3.7296E+03	3.1954E+03
	Avg	3.9921E+03	3.3419E+03	3.8750E+03	3.3033E+03
	Std	8.6555E+01	4.2269E+01	6.7595E+01	6.0247E+01
F24	Best	4.4612E+03	3.6671E+03	4.3180E+03	3.6717E+03
	Avg	4.5970E+03	3.8102E+03	4.4934E+03	3.7765E+03
	Std	9.2196E+01	7.8570E+01	9.0759E+01	6.9660E+01
F25	Best	5.5350E+03	3.8894E+03	4.6325E+03	3.8613E+03
	Avg	6.4021E+03	4.5753E+03	5.4308E+03	4.1119E+03
	Std	5.9407E+02	3.9784E+02	5.0231E+02	2.3349E+02
F26	Best	1.7376E+04	9.6698E+03	1.6441E+04	9.5383E+03
	Avg	1.8534E+04	1.1703E+04	1.7623E+04	1.0801E+04
	Std	8.0071E+02	1.2552E+03	6.6396E+02	7.3785E+02
F27	Best	4.1218E+03	3.5785E+03	3.8921E+03	3.5825E+03
	Avg	4.3584E+03	3.7467E+03	4.0526E+03	3.7640E+03
	Std	1.5682E+02	1.1397E+02	1.1497E+02	1.4854E+02
F28	Best	7.4043E+03	4.2100E+03	5.4099E+03	4.2733E+03
	Avg	8.6495E+03	5.5227E+03	6.9081E+03	5.0133E+03
	Std	7.2869E+02	9.2520E+02	1.0707E+03	6.2226E+02
F29	Best	9.6161E+03	6.8033E+03	8.7405E+03	6.6645E+03
	Avg	1.1001E+04	7.7497E+03	9.7084E+03	7.4722E+03
	Std	8.5091E+02	3.8334E+02	4.2583E+02	4.8964E+02
F30	Best	1.9568E+08	9.2708E+06	3.4986E+07	5.9545E+06
	Avg	3.6528E+08	2.4810E+07	6.9686E+07	1.6935E+07
	Std	1.4347E+08	1.0887E+07	3.0132E+07	9.2897E+06

**Table A5:** The statistical results of MESBOA and comparison algorithms on the CEC 2022 in dimensions of 10.

No.	Index	MESBOA	SBOA	AE	LSHADE-SPACMA	EO	GLS-RIME	CFOA	ISGTOA	RBMO	ESLPSO
F1	Min	3.0008E+02	5.5352E+02	5.1390E+02	3.0422E+02	8.3144E+02	3.2002E+02	4.2691E+02	8.9581E+02	5.3312E+02	1.4101E+03
	Avg	3.1454E+02	1.4071E+03	8.0759E+02	3.3737E+02	2.9846E+03	5.1977E+02	1.7090E+03	2.0241E+03	1.3024E+03	2.7115E+03
	Std	1.3722E+01	6.0831E+02	2.2829E+02	5.0808E+01	1.6190E+03	2.0288E+02	7.7035E+02	1.1310E+03	5.5710E+02	8.2496E+02
F2	Min	4.0086E+02	4.0601E+02	4.0003E+02	4.0049E+02	4.0762E+02	4.0054E+02	4.0266E+02	4.0006E+02	4.0055E+02	4.0026E+02
	Avg	4.1401E+02	4.1381E+02	4.0334E+02	4.0680E+02	4.1322E+02	4.1070E+02	4.2983E+02	4.1291E+02	4.1438E+02	4.0746E+02
	Std	1.9802E+01	1.5470E+01	3.6336E+00	2.0036E+00	1.1389E+01	1.6317E+01	2.2947E+01	1.9173E+01	1.9443E+01	2.4406E+00
F3	Min	6.0009E+02	6.0092E+02	6.0010E+02	6.0079E+02	6.0047E+02	6.0029E+02	6.0831E+02	6.0023E+02	6.0029E+02	6.0027E+02
	Avg	6.0021E+02	6.0350E+02	6.0022E+02	6.0131E+02	6.0149E+02	6.0168E+02	6.1368E+02	6.0083E+02	6.0133E+02	6.0052E+02
	Std	7.1382E−02	1.3381E+00	7.7883E−02	3.1576E−01	7.5906E−01	8.3931E−01	3.2778E+00	4.1407E−01	1.1305E+00	1.2418E−01
F4	Min	8.0200E+02	8.0797E+02	8.0629E+02	8.2205E+02	8.0617E+02	8.0931E+02	8.0659E+02	8.1427E+02	8.0465E+02	8.2239E+02
	Avg	8.1592E+02	8.2306E+02	8.2573E+02	8.3256E+02	8.1799E+02	8.2125E+02	8.1999E+02	8.3406E+02	8.2102E+02	8.3557E+02
	Std	6.6586E+00	7.5038E+00	7.9831E+00	5.3833E+00	5.9919E+00	5.8839E+00	7.9805E+00	8.3771E+00	9.6320E+00	5.5557E+00
F5	Min	9.0000E+02	9.0120E+02	9.0001E+02	9.0017E+02	9.0025E+02	9.0037E+02	9.1103E+02	9.0009E+02	9.0009E+02	9.0009E+02
	Avg	9.0009E+02	9.0615E+02	9.0004E+02	9.0095E+02	9.0394E+02	9.0513E+02	9.6060E+02	9.0055E+02	9.0440E+02	9.0016E+02
	Std	1.0028E−01	5.0025E+00	5.0214E−02	6.4519E−01	6.5938E+00	6.6883E+00	3.3360E+01	4.7008E−01	4.9037E+00	5.0318E−02
F6	Min	1.9415E+03	6.5012E+03	1.9615E+03	1.8758E+03	1.9105E+03	1.9400E+03	1.8877E+03	2.2054E+03	2.3819E+03	3.0471E+03
	Avg	4.0973E+03	5.3346E+04	3.1572E+03	2.0856E+03	5.3879E+03	7.6145E+03	3.5093E+03	1.0229E+04	6.4031E+03	9.9574E+03
	Std	1.9009E+03	4.1672E+04	1.0221E+03	1.3273E+02	2.4792E+03	1.0724E+04	1.6457E+03	7.5371E+03	4.2630E+03	5.0076E+03
F7	Min	2.0173E+03	2.0198E+03	2.0263E+03	2.0245E+03	2.0138E+03	2.0119E+03	2.0202E+03	2.0291E+03	2.0224E+03	2.0270E+03
	Avg	2.0237E+03	2.0346E+03	2.0424E+03	2.0376E+03	2.0266E+03	2.0245E+03	2.0410E+03	2.0405E+03	2.0291E+03	2.0395E+03
	Std	2.9332E+00	6.9307E+00	7.0220E+00	5.4251E+00	4.9229E+00	4.5714E+00	9.4837E+00	6.4143E+00	6.0680E+00	6.3562E+00
F8	Min	2.2020E+03	2.2170E+03	2.2247E+03	2.2193E+03	2.2122E+03	2.2058E+03	2.2215E+03	2.2219E+03	2.2109E+03	2.2143E+03
	Avg	2.2213E+03	2.2280E+03	2.2298E+03	2.2276E+03	2.2269E+03	2.2238E+03	2.2269E+03	2.2310E+03	2.2260E+03	2.2285E+03
	Std	6.3528E+00	3.9364E+00	1.9205E+00	2.1586E+00	3.7518E+00	4.9446E+00	3.1650E+00	3.1939E+00	5.0430E+00	3.8392E+00
F9	Min	2.5293E+03	2.5294E+03	2.5293E+03	2.5293E+03	2.5293E+03	2.5293E+03	2.5300E+03	2.5293E+03	2.5293E+03	2.5293E+03
	Avg	2.5293E+03	2.5300E+03	2.5294E+03	2.5293E+03	2.5313E+03	2.5299E+03	2.5485E+03	2.5295E+03	2.5306E+03	2.5293E+03
	Std	2.8182E−02	5.5915E−01	7.6917E−02	2.2856E−02	3.4659E+00	1.0896E+00	1.6445E+01	2.7927E−01	6.8452E+00	1.5645E−03
F10	Min	2.5003E+03	2.5004E+03	2.5004E+03	2.5004E+03	2.5002E+03	2.5003E+03	2.5004E+03	2.5004E+03	2.5003E+03	2.5003E+03
	Avg	2.5155E+03	2.5217E+03	2.5005E+03	2.5005E+03	2.5239E+03	2.5121E+03	2.5014E+03	2.5284E+03	2.5162E+03	2.5279E+03
	Std	3.9134E+01	4.7554E+01	7.8479E−02	7.9151E−02	4.7604E+01	3.5340E+01	2.0170E+00	5.1316E+01	4.0826E+01	5.5774E+01
F11	Min	2.6004E+03	2.6578E+03	2.9200E+03	2.6417E+03	2.7296E+03	2.6739E+03	2.7942E+03	2.6411E+03	2.7474E+03	2.6188E+03
	Avg	2.7765E+03	2.8522E+03	2.9269E+03	2.7351E+03	2.9018E+03	2.9883E+03	3.5609E+03	2.8197E+03	2.9310E+03	2.8669E+03
	Std	1.7604E+02	1.4014E+02	5.1499E+00	1.1136E+02	1.3348E+02	1.3768E+02	2.8950E+02	1.1526E+02	1.7076E+02	1.1558E+02
F12	Min	2.8607E+03	2.8611E+03	2.8642E+03	2.8628E+03	2.8626E+03	2.8597E+03	2.8634E+03	2.8611E+03	2.8586E+03	2.8594E+03
	Avg	2.8632E+03	2.8641E+03	2.8649E+03	2.8645E+03	2.8645E+03	2.8649E+03	2.8673E+03	2.8638E+03	2.8635E+03	2.8631E+03
	Std	1.1067E+00	9.7370E−01	1.7525E−01	6.9615E−01	1.0259E+00	1.7099E+00	2.1460E+00	1.1296E+00	1.9248E+00	1.5283E+00

**Table A6:** The statistical results of MESBOA and comparison algorithms on the CEC 2022 in dimensions of 20.

No.	Index	MESBOA	SBOA	AE	LSHADE-SPACMA	EO	GLS-RIME	CFOA	ISGTOA	RBMO	ESLPSO
F1	Min	8.3858E+02	5.0359E+03	5.0137E+03	4.8345E+02	6.0819E+03	1.8946E+03	6.0716E+03	9.4921E+03	6.1834E+03	1.0408E+04
	Avg	1.6891E+03	1.4221E+04	1.0012E+04	3.7849E+03	1.8233E+04	7.5399E+03	1.1330E+04	1.5542E+04	1.3538E+04	1.6375E+04
	Std	6.9714E+02	5.1642E+03	2.7528E+03	7.1336E+03	8.4061E+03	3.5894E+03	3.0172E+03	3.4222E+03	4.0194E+03	3.6280E+03
F2	Min	4.1915E+02	4.5978E+02	4.4998E+02	4.4534E+02	4.6123E+02	4.4764E+02	5.9380E+02	4.3119E+02	4.4737E+02	4.4746E+02
	Avg	4.5938E+02	5.0292E+02	4.5559E+02	4.4917E+02	4.9334E+02	4.6999E+02	6.8437E+02	4.7549E+02	4.8875E+02	4.5052E+02
	Std	1.3063E+01	4.3153E+01	6.0910E+00	9.0422E−01	2.1541E+01	2.1780E+01	4.4410E+01	2.2747E+01	4.7006E+01	4.6467E+00
F3	Min	6.0053E+02	6.0755E+02	6.0085E+02	6.0219E+02	6.0302E+02	6.0182E+02	6.2440E+02	6.0114E+02	6.0295E+02	6.0195E+02
	Avg	6.0128E+02	6.1309E+02	6.0126E+02	6.0291E+02	6.0536E+02	6.0784E+02	6.3605E+02	6.0467E+02	6.0941E+02	6.0272E+02
	Std	5.9607E−01	4.4226E+00	3.4668E−01	3.7870E−01	1.6710E+00	3.7469E+00	6.1865E+00	1.8189E+00	4.8050E+00	3.8536E−01
F4	Min	8.0729E+02	8.5622E+02	8.5720E+02	8.6587E+02	8.3745E+02	8.3555E+02	8.5345E+02	8.7606E+02	8.4342E+02	8.9304E+02
	Avg	8.4025E+02	8.9906E+02	8.9627E+02	9.0914E+02	8.6187E+02	8.6109E+02	8.8274E+02	9.1074E+02	8.7786E+02	9.1004E+02
	Std	1.4961E+01	1.3812E+01	1.1648E+01	1.0702E+01	1.3602E+01	1.6565E+01	1.3996E+01	1.8822E+01	1.9546E+01	9.5969E+00
F5	Min	9.0177E+02	9.7435E+02	9.0053E+02	9.0410E+02	9.0999E+02	9.2266E+02	1.2693E+03	9.0312E+02	9.2617E+02	9.0257E+02
	Avg	9.2697E+02	1.1351E+03	9.0196E+02	9.0933E+02	9.8628E+02	1.3849E+03	1.6357E+03	9.3639E+02	1.1046E+03	9.0606E+02
	Std	2.7455E+01	1.5742E+02	1.5708E+00	3.4103E+00	1.1422E+02	7.3379E+02	2.1302E+02	3.3885E+01	1.5536E+02	1.6011E+00
F6	Min	2.4415E+03	5.6861E+05	9.2025E+03	2.9828E+04	1.1292E+04	1.8234E+04	3.2038E+03	1.3510E+04	2.9496E+03	2.6406E+05
	Avg	1.6519E+04	3.2073E+06	2.2559E+04	7.5818E+04	7.1584E+04	1.3934E+05	1.5325E+06	5.7316E+04	1.5054E+04	8.8550E+05
	Std	1.4728E+04	2.7864E+06	1.0145E+04	3.8215E+04	5.8190E+04	1.1645E+05	2.7417E+06	5.6502E+04	1.2007E+04	4.2928E+05
F7	Min	2.0314E+03	2.0573E+03	2.0906E+03	2.1036E+03	2.0472E+03	2.0302E+03	2.0733E+03	2.0685E+03	2.0516E+03	2.0849E+03
	Avg	2.0525E+03	2.0973E+03	2.1135E+03	2.1237E+03	2.0724E+03	2.0676E+03	2.1074E+03	2.1090E+03	2.0843E+03	2.1202E+03
	Std	1.5269E+01	1.7843E+01	1.2073E+01	1.2044E+01	1.4800E+01	2.3012E+01	1.9913E+01	2.0604E+01	2.6711E+01	1.3455E+01
F8	Min	2.2239E+03	2.2315E+03	2.2325E+03	2.2343E+03	2.2295E+03	2.2252E+03	2.2265E+03	2.2365E+03	2.2302E+03	2.2355E+03
	Avg	2.2297E+03	2.2426E+03	2.2386E+03	2.2425E+03	2.2401E+03	2.2359E+03	2.2438E+03	2.2455E+03	2.2429E+03	2.2440E+03
	Std	4.0834E+00	6.2336E+00	3.4048E+00	3.6483E+00	2.3137E+01	9.0629E+00	3.2767E+01	5.4467E+00	2.1835E+01	4.8815E+00
F9	Min	2.4809E+03	2.4844E+03	2.4816E+03	2.4809E+03	2.4825E+03	2.4832E+03	2.4966E+03	2.4813E+03	2.4810E+03	2.4810E+03
	Avg	2.4820E+03	2.4926E+03	2.4823E+03	2.4810E+03	2.4904E+03	2.4870E+03	2.5420E+03	2.4833E+03	2.4863E+03	2.4813E+03
	Std	1.2667E+00	7.4879E+00	4.4357E−01	3.7147E−02	5.6361E+00	3.7420E+00	2.3935E+01	1.1033E+00	8.0031E+00	2.2626E−01
F10	Min	2.5005E+03	2.5006E+03	2.5005E+03	2.5006E+03	2.5004E+03	2.5004E+03	2.5014E+03	2.5005E+03	2.5005E+03	2.5005E+03
	Avg	2.6017E+03	2.5296E+03	2.5008E+03	2.5009E+03	3.0286E+03	2.7275E+03	2.5347E+03	2.7729E+03	3.1864E+03	2.6536E+03
	Std	3.1332E+02	7.3987E+01	1.5152E−01	1.5282E−01	1.0559E+03	3.4159E+02	6.9079E+01	9.5919E+02	1.1463E+03	7.2065E+02
F11	Min	2.9085E+03	3.2248E+03	3.1909E+03	3.0928E+03	3.3452E+03	3.2947E+03	5.2399E+03	3.0214E+03	4.0363E+03	3.2159E+03
	Avg	2.9622E+03	3.4671E+03	3.2899E+03	3.1500E+03	3.7130E+03	3.8003E+03	6.2917E+03	3.1761E+03	5.1874E+03	3.3070E+03
	Std	5.4120E+01	1.4417E+02	5.0259E+01	3.5659E+01	2.0350E+02	2.4286E+02	6.0621E+02	9.0105E+01	6.6941E+02	4.4862E+01
F12	Min	2.9370E+03	2.9452E+03	2.9493E+03	2.9437E+03	2.9450E+03	2.9425E+03	2.9657E+03	2.9438E+03	2.9445E+03	2.9375E+03
	Avg	2.9526E+03	2.9608E+03	2.9587E+03	2.9487E+03	2.9570E+03	2.9639E+03	3.0023E+03	2.9527E+03	2.9653E+03	2.9478E+03
	Std	1.2159E+01	1.4357E+01	5.4247E+00	3.5889E+00	9.0443E+00	1.4519E+01	2.2120E+01	7.4852E+00	1.9629E+01	4.3820E+00

**Table A7:** The statistical results of MESBOA and comparison algorithms on the CEC 2017 in dimensions of 50.

No.	Index	MESBOA	SBOA	AE	LSHADE-SPACMA	EO	GLS-RIME	CFOA	ISGTOA	RBMO	ESLPSO
F1	Min	1.1637E+07	4.7764E+09	1.6137E+08	2.2525E+07	3.5383E+09	6.4069E+08	3.4167E+10	2.8046E+08	5.2282E+09	3.1555E+08
	Avg	2.2657E+08	6.9499E+09	2.5288E+08	2.9953E+07	6.4823E+09	2.0564E+09	3.9688E+10	8.0049E+08	9.4480E+09	4.0986E+08
	Std	2.5481E+08	1.5315E+09	5.6996E+07	3.9012E+06	1.6721E+09	6.5130E+08	4.1088E+09	3.0618E+08	2.0420E+09	4.6561E+07
F3	Min	5.5114E+04	1.1419E+05	1.1382E+05	3.2605E+04	8.4462E+04	9.4133E+04	8.4171E+04	1.0376E+05	1.0619E+05	1.2185E+05
	Avg	8.3334E+04	1.5803E+05	1.4165E+05	9.6727E+04	1.9109E+05	1.3644E+05	1.0696E+05	1.2448E+05	1.6364E+05	1.6084E+05
	Std	1.4144E+04	2.9204E+04	1.4019E+04	5.3375E+04	4.7511E+04	3.0109E+04	1.4652E+04	1.2106E+04	3.3896E+04	2.0252E+04
F4	Min	5.8204E+02	9.0607E+02	6.7578E+02	6.0897E+02	8.2145E+02	6.9497E+02	5.0992E+03	6.1053E+02	8.4719E+02	6.4135E+02
	Avg	6.9853E+02	1.2153E+03	7.1358E+02	6.3199E+02	1.0857E+03	8.1578E+02	6.9549E+03	7.6741E+02	1.4115E+03	6.8002E+02
	Std	7.8234E+01	1.8166E+02	1.9092E+01	7.6351E+00	1.5089E+02	8.1195E+01	1.0882E+03	7.4666E+01	4.1504E+02	1.6677E+01
F5	Min	6.0263E+02	8.6957E+02	7.9146E+02	8.3611E+02	7.5363E+02	7.0390E+02	8.5361E+02	8.5835E+02	7.6732E+02	8.6251E+02
	Avg	6.5415E+02	9.3966E+02	8.8352E+02	8.6352E+02	8.0003E+02	8.1591E+02	9.2136E+02	9.2996E+02	8.3821E+02	8.8635E+02
	Std	3.2748E+01	4.2751E+01	2.7514E+01	1.3759E+01	2.6494E+01	4.3410E+01	3.3014E+01	3.4180E+01	3.9892E+01	1.5935E+01
F6	Min	6.0782E+02	6.3033E+02	6.0496E+02	6.0372E+02	6.1387E+02	6.1487E+02	6.5337E+02	6.1919E+02	6.2426E+02	6.0857E+02
	Avg	6.1286E+02	6.4276E+02	6.0638E+02	6.0434E+02	6.2064E+02	6.2835E+02	6.6474E+02	6.2636E+02	6.3294E+02	6.1010E+02
	Std	3.1206E+00	7.0015E+00	7.9083E−01	3.8074E−01	3.3924E+00	8.0356E+00	7.0467E+00	5.3859E+00	7.5354E+00	6.6633E−01
F7	Min	8.9606E+02	1.2198E+03	1.1172E+03	1.0994E+03	1.0967E+03	1.1388E+03	1.2987E+03	1.2021E+03	1.1585E+03	1.1135E+03
	Avg	9.6889E+02	1.3159E+03	1.1641E+03	1.1261E+03	1.1470E+03	1.2772E+03	1.4415E+03	1.2516E+03	1.3285E+03	1.1534E+03
	Std	5.1402E+01	6.1956E+01	1.9926E+01	1.5580E+01	2.9420E+01	1.0871E+02	5.2585E+01	3.5744E+01	9.9671E+01	1.6449E+01
F8	Min	8.9896E+02	1.2207E+03	1.0897E+03	1.1207E+03	1.0674E+03	1.0245E+03	1.1777E+03	1.1738E+03	1.0705E+03	1.1640E+03
	Avg	9.5861E+02	1.2694E+03	1.1767E+03	1.1670E+03	1.1083E+03	1.1040E+03	1.2432E+03	1.2420E+03	1.1381E+03	1.1940E+03
	Std	2.6639E+01	3.2613E+01	2.9062E+01	1.8112E+01	2.6509E+01	3.9981E+01	2.4802E+01	2.7793E+01	3.6529E+01	1.1745E+01
F9	Min	1.8049E+03	6.6165E+03	1.1067E+03	9.5472E+02	3.3378E+03	3.7653E+03	1.0706E+04	3.0292E+03	4.5584E+03	1.2680E+03
	Avg	2.6398E+03	1.4808E+04	1.2845E+03	9.7584E+02	4.9030E+03	8.5219E+03	1.5726E+04	7.1289E+03	1.0015E+04	1.3741E+03
	Std	5.4029E+02	5.7247E+03	1.1266E+02	1.7308E+01	1.4975E+03	3.8088E+03	3.3257E+03	2.1415E+03	4.6663E+03	1.0687E+02
F10	Min	6.6141E+03	1.3360E+04	1.3614E+04	1.4242E+04	1.1002E+04	9.0746E+03	1.0091E+04	1.2830E+04	1.1341E+04	1.3925E+04
	Avg	7.6110E+03	1.4135E+04	1.4722E+04	1.4948E+04	1.2475E+04	1.0175E+04	1.1493E+04	1.4741E+04	1.3954E+04	1.5116E+04
	Std	8.0063E+02	5.2760E+02	5.0009E+02	3.5680E+02	7.0032E+02	8.6858E+02	9.2976E+02	7.8651E+02	9.9558E+02	4.4676E+02
F11	Min	1.3298E+03	2.2508E+03	2.4813E+03	1.3972E+03	2.5198E+03	1.7715E+03	3.6685E+03	4.0055E+03	2.4331E+03	1.6679E+03
	Avg	1.4711E+03	3.0593E+03	2.8762E+03	1.4551E+03	3.1720E+03	2.1821E+03	7.2863E+03	6.2807E+03	3.1983E+03	1.7964E+03
	Std	1.0587E+02	4.8769E+02	2.5777E+02	3.1538E+01	5.5770E+02	1.8117E+02	2.6144E+03	1.6766E+03	4.4591E+02	1.0066E+02
F12	Min	6.1424E+06	4.1831E+08	1.8240E+07	7.4816E+06	6.4276E+07	1.8047E+08	5.2475E+09	3.5798E+07	1.0240E+08	5.5660E+07
	Avg	4.1849E+07	9.7543E+08	3.3449E+07	1.2350E+07	3.1236E+08	4.4457E+08	8.6908E+09	8.0726E+07	4.3543E+08	8.2098E+07
	Std	2.3945E+07	3.5801E+08	8.7471E+06	2.2153E+06	2.0020E+08	2.8385E+08	2.5393E+09	2.9773E+07	3.1062E+08	1.7336E+07
F13	Min	5.2040E+04	2.2119E+07	2.3060E+05	9.3286E+05	1.0703E+06	2.6648E+06	3.5431E+08	2.0570E+05	5.2995E+05	1.9709E+06
	Avg	1.8121E+05	9.4019E+07	5.3652E+05	1.3091E+06	4.2206E+06	1.0790E+07	1.2022E+09	4.7791E+05	1.8467E+06	4.2753E+06
	Std	1.8183E+05	5.3054E+07	1.4560E+05	2.5627E+05	3.0616E+06	6.4087E+06	6.8556E+08	2.0227E+05	1.2132E+06	1.8237E+06
F14	Min	1.2565E+04	2.2017E+05	5.5061E+04	7.0420E+03	1.2522E+05	1.1014E+05	1.4299E+05	6.0862E+04	6.0021E+04	2.0606E+04
	Avg	5.9156E+04	8.6151E+05	1.4746E+05	1.1116E+04	6.7541E+05	6.0659E+05	9.8572E+05	3.9105E+05	3.0145E+05	5.8547E+04
	Std	4.2589E+04	5.9003E+05	6.0540E+04	3.0263E+03	5.7999E+05	3.7000E+05	6.9025E+05	2.4357E+05	1.8631E+05	3.0753E+04
F15	Min	1.8875E+04	1.2095E+06	3.3517E+04	1.1288E+05	3.7597E+04	1.7350E+05	6.8299E+05	2.9623E+04	2.5477E+04	2.8704E+05
	Avg	4.7521E+04	6.1467E+06	6.7053E+04	2.9669E+05	1.5101E+05	1.9413E+06	6.9673E+07	5.8664E+04	1.3149E+05	8.2116E+05
	Std	2.1867E+04	4.8189E+06	2.3812E+04	1.0654E+05	1.6007E+05	2.0669E+06	6.9288E+07	2.2445E+04	1.1291E+05	4.3197E+05
F16	Min	2.4956E+03	3.9486E+03	4.0112E+03	4.3743E+03	2.4226E+03	3.0663E+03	3.4434E+03	3.2962E+03	3.1865E+03	4.2754E+03
	Avg	3.2651E+03	4.6951E+03	4.4243E+03	4.7903E+03	3.4440E+03	3.7077E+03	4.1991E+03	4.3823E+03	3.8493E+03	4.8817E+03
	Std	4.9977E+02	3.8900E+02	2.0462E+02	1.9834E+02	5.6927E+02	3.6016E+02	3.5669E+02	5.4907E+02	3.8406E+02	2.3734E+02
F17	Min	2.3661E+03	3.0421E+03	3.3599E+03	3.5177E+03	2.6689E+03	2.9253E+03	3.3796E+03	2.8986E+03	2.5455E+03	3.3567E+03
	Avg	2.8727E+03	3.7092E+03	3.7582E+03	3.8154E+03	3.0464E+03	3.4316E+03	3.7957E+03	3.8118E+03	3.3002E+03	3.7886E+03
	Std	2.2240E+02	3.1256E+02	1.7578E+02	1.6307E+02	2.3491E+02	3.3808E+02	3.3379E+02	3.5180E+02	3.7505E+02	2.0355E+02
F18	Min	1.5981E+05	1.5684E+06	9.9232E+05	8.0688E+04	1.3778E+06	7.6559E+05	8.7248E+05	9.2366E+05	6.9114E+05	2.0764E+05
	Avg	9.3977E+05	6.1094E+06	1.8558E+06	1.2446E+05	5.6208E+06	3.7015E+06	4.7521E+06	3.9587E+06	3.9771E+06	5.3601E+05
	Std	8.0500E+05	3.4628E+06	4.2155E+05	3.0518E+04	3.5134E+06	3.5382E+06	3.1706E+06	2.6464E+06	2.3269E+06	2.6820E+05
F19	Min	4.4091E+03	1.3915E+06	5.6298E+04	4.4950E+04	6.5644E+04	2.1257E+05	8.1248E+05	5.7614E+04	4.6500E+04	1.3341E+05
	Avg	2.6545E+04	4.4259E+06	1.0510E+05	1.2504E+05	3.6678E+05	3.2405E+06	3.5260E+07	1.1431E+05	9.1958E+05	4.9753E+05
	Std	1.8174E+04	3.9654E+06	2.9584E+04	3.5744E+04	2.2511E+05	2.6170E+06	4.5132E+07	4.1283E+04	2.5004E+06	3.0733E+05
F20	Min	2.6254E+03	3.1774E+03	3.5112E+03	3.7067E+03	2.5335E+03	2.7123E+03	2.7964E+03	3.1285E+03	2.8483E+03	3.6638E+03
	Avg	3.0731E+03	3.7160E+03	3.8384E+03	3.9985E+03	3.0484E+03	3.2575E+03	3.3176E+03	3.8956E+03	3.7055E+03	4.0315E+03
	Std	2.5375E+02	2.7517E+02	1.5287E+02	1.3770E+02	2.3416E+02	2.5920E+02	2.7126E+02	2.8947E+02	3.3768E+02	1.7560E+02
F21	Min	2.3792E+03	2.6786E+03	2.5625E+03	2.6492E+03	2.5543E+03	2.5328E+03	2.7195E+03	2.6732E+03	2.5859E+03	2.6567E+03
	Avg	2.4544E+03	2.7511E+03	2.6587E+03	2.6685E+03	2.6011E+03	2.5884E+03	2.7729E+03	2.7221E+03	2.6344E+03	2.6871E+03
	Std	3.9283E+01	3.7449E+01	2.8911E+01	1.2349E+01	2.7354E+01	3.4588E+01	4.0765E+01	2.6015E+01	3.9733E+01	1.3573E+01
F22	Min	2.4725E+03	3.3486E+03	3.5921E+03	1.4086E+04	1.2030E+04	9.6829E+03	1.1464E+04	1.4865E+04	1.2941E+04	1.5649E+04
	Avg	9.2499E+03	1.2546E+04	1.4352E+04	1.6008E+04	1.3742E+04	1.1532E+04	1.3372E+04	1.6342E+04	1.5052E+04	1.6525E+04
	Std	1.8300E+03	5.3364E+03	4.0623E+03	9.6707E+02	8.7931E+02	1.1332E+03	7.6100E+02	5.9168E+02	1.0216E+03	4.0376E+02
F23	Min	2.8376E+03	3.1260E+03	3.0431E+03	3.0736E+03	2.9691E+03	2.9620E+03	3.2427E+03	3.0853E+03	3.0483E+03	3.0751E+03
	Avg	2.8974E+03	3.2004E+03	3.0969E+03	3.1028E+03	3.0301E+03	3.0724E+03	3.3675E+03	3.1760E+03	3.1240E+03	3.1190E+03
	Std	4.2283E+01	3.7234E+01	2.6677E+01	1.3174E+01	3.0509E+01	7.0376E+01	6.8208E+01	4.0798E+01	5.1688E+01	1.8247E+01
F24	Min	2.9859E+03	3.2792E+03	3.1316E+03	3.2340E+03	3.1426E+03	3.1259E+03	3.4064E+03	3.2570E+03	3.1614E+03	3.2434E+03
	Avg	3.0375E+03	3.3415E+03	3.2318E+03	3.2608E+03	3.1886E+03	3.1904E+03	3.5290E+03	3.3190E+03	3.2688E+03	3.2777E+03
	Std	3.1315E+01	4.6955E+01	4.5076E+01	1.4964E+01	2.2587E+01	5.1510E+01	9.1840E+01	4.3127E+01	5.5818E+01	1.5457E+01
F25	Min	3.0471E+03	3.4077E+03	3.1609E+03	3.0330E+03	3.4203E+03	3.1473E+03	4.8272E+03	3.1928E+03	3.5785E+03	3.1017E+03
	Avg	3.1542E+03	3.5906E+03	3.1995E+03	3.0556E+03	3.6879E+03	3.3543E+03	6.6008E+03	3.2818E+03	3.9623E+03	3.1461E+03
	Std	6.0750E+01	1.2272E+02	2.4260E+01	7.5154E+00	1.8336E+02	1.2482E+02	7.1334E+02	6.1400E+01	2.6218E+02	3.4903E+01
F26	Min	4.7051E+03	5.1018E+03	6.4118E+03	6.9767E+03	6.0872E+03	6.4303E+03	9.8786E+03	4.0135E+03	7.1171E+03	7.2342E+03
	Avg	5.4806E+03	8.2003E+03	7.3411E+03	7.2807E+03	6.8866E+03	7.3844E+03	1.1680E+04	7.8856E+03	7.8647E+03	7.5074E+03
	Std	4.3395E+02	1.0809E+03	3.2503E+02	1.4417E+02	3.6657E+02	6.5109E+02	9.8412E+02	1.0214E+03	5.7280E+02	1.7217E+02
F27	Min	3.3330E+03	3.4221E+03	3.4397E+03	3.2776E+03	3.4577E+03	3.4426E+03	3.7015E+03	3.4066E+03	3.4035E+03	3.3536E+03
	Avg	3.4430E+03	3.5984E+03	3.5395E+03	3.3168E+03	3.5731E+03	3.5522E+03	4.0335E+03	3.5534E+03	3.5512E+03	3.4254E+03
	Std	7.9216E+01	1.0979E+02	5.8002E+01	2.3753E+01	8.9246E+01	9.0290E+01	1.5058E+02	1.0113E+02	6.6937E+01	5.0257E+01
F28	Min	3.3525E+03	3.7182E+03	3.4353E+03	3.3079E+03	4.0901E+03	3.5024E+03	5.9581E+03	3.5314E+03	3.7771E+03	3.3317E+03
	Avg	3.4983E+03	4.0394E+03	3.5413E+03	3.3332E+03	4.5248E+03	3.9023E+03	6.7727E+03	3.7508E+03	5.2794E+03	3.4113E+03
	Std	8.8663E+01	2.7281E+02	4.1650E+01	1.1294E+01	2.9426E+02	3.4787E+02	3.7421E+02	1.5303E+02	1.0948E+03	4.1444E+01
F29	Min	3.9684E+03	4.8407E+03	4.7729E+03	4.6065E+03	3.8375E+03	4.2339E+03	5.6545E+03	4.4793E+03	4.6342E+03	4.8666E+03
	Avg	4.4064E+03	5.6600E+03	5.2761E+03	4.9558E+03	4.5596E+03	4.9037E+03	6.9154E+03	5.2085E+03	5.2821E+03	5.2969E+03
	Std	2.8843E+02	5.6403E+02	2.2883E+02	1.6150E+02	3.6955E+02	3.1961E+02	8.3565E+02	4.4779E+02	3.3418E+02	2.3115E+02
F30	Min	5.2299E+06	3.2291E+07	1.1142E+07	5.2184E+06	2.6677E+07	3.0475E+07	6.1540E+07	1.9720E+07	2.1650E+07	1.9344E+07
	Avg	1.2025E+07	9.7919E+07	1.4284E+07	6.6462E+06	4.8370E+07	8.4366E+07	2.0725E+08	3.2490E+07	4.3124E+07	3.0945E+07
	Std	5.1684E+06	4.8821E+07	2.0853E+06	1.0543E+06	1.4954E+07	4.1446E+07	9.5221E+07	7.3858E+06	2.4642E+07	6.4771E+06

**Table A8:** The statistical results of MESBOA and comparison algorithms on the CEC 2017 in dimensions of 100.

No.	Index	MESBOA	SBOA	AE	LSHADE-SPACMA	EO	GLS-RIME	CFOA	ISGTOA	RBMO	ESLPSO
F1	Min	4.7578E+08	2.7965E+10	2.7742E+09	1.6279E+08	4.0603E+10	1.7793E+10	1.2251E+11	8.1416E+09	5.6575E+10	3.6241E+09
	Avg	5.6775E+09	4.2303E+10	3.6002E+09	2.2889E+08	5.6506E+10	2.5450E+10	1.4282E+11	1.5226E+10	7.6170E+10	4.1816E+09
	Std	2.5400E+09	7.5606E+09	4.9040E+08	3.7658E+07	8.4391E+09	5.4912E+09	8.8983E+09	2.8838E+09	1.0757E+10	4.3519E+08
F3	Min	2.4978E+05	3.3304E+05	2.9931E+05	2.7546E+05	3.5957E+05	3.4439E+05	2.4726E+05	2.3127E+05	3.1610E+05	3.0301E+05
	Avg	2.9947E+05	4.1536E+05	3.3198E+05	4.1539E+05	5.0835E+05	4.5767E+05	2.7484E+05	3.0991E+05	4.0874E+05	3.9833E+05
	Std	3.1702E+04	6.1233E+04	1.2068E+04	8.1002E+04	7.3109E+04	6.8895E+04	1.9099E+04	2.7953E+04	7.2435E+04	4.1742E+04
F4	Min	1.0915E+03	3.7713E+03	1.2507E+03	7.3749E+02	3.3652E+03	2.5327E+03	1.8432E+04	1.7752E+03	6.4140E+03	1.1556E+03
	Avg	1.4961E+03	4.6009E+03	1.3401E+03	7.7483E+02	4.9072E+03	3.3766E+03	2.5173E+04	2.2301E+03	8.8521E+03	1.3561E+03
	Std	2.4387E+02	5.5424E+02	4.7487E+01	1.4339E+01	9.6920E+02	7.4737E+02	4.0788E+03	2.7114E+02	1.6907E+03	9.8565E+01
F5	Min	8.5788E+02	1.5227E+03	1.3573E+03	1.2564E+03	1.2389E+03	1.2027E+03	1.5845E+03	1.3602E+03	1.3539E+03	1.3879E+03
	Avg	9.6856E+02	1.6441E+03	1.4547E+03	1.3137E+03	1.3756E+03	1.4159E+03	1.6584E+03	1.6007E+03	1.4718E+03	1.4237E+03
	Std	7.9858E+01	8.0152E+01	4.1082E+01	2.7698E+01	6.2044E+01	8.9103E+01	4.4592E+01	8.4973E+01	7.2580E+01	2.2443E+01
F6	Min	6.2450E+02	6.5576E+02	6.1200E+02	6.0498E+02	6.3638E+02	6.3858E+02	6.7517E+02	6.4310E+02	6.4781E+02	6.1690E+02
	Avg	6.3054E+02	6.6823E+02	6.1477E+02	6.0555E+02	6.4365E+02	6.5297E+02	6.8155E+02	6.5437E+02	6.5485E+02	6.1883E+02
	Std	3.4382E+00	7.0838E+00	1.6297E+00	3.3688E−01	5.1255E+00	7.7214E+00	3.6274E+00	6.0821E+00	4.7458E+00	1.0117E+00
F7	Min	1.3484E+03	2.2647E+03	1.6787E+03	1.5984E+03	2.0042E+03	2.1229E+03	2.6854E+03	2.0861E+03	2.4234E+03	1.7324E+03
	Avg	1.6036E+03	2.5394E+03	1.7307E+03	1.6411E+03	2.2239E+03	2.4803E+03	3.0084E+03	2.2079E+03	2.8694E+03	1.7951E+03
	Std	9.9635E+01	1.2114E+02	2.9692E+01	1.9611E+01	9.9119E+01	2.1180E+02	1.1779E+02	7.0756E+01	2.6005E+02	3.2300E+01
F8	Min	1.1617E+03	1.8758E+03	1.7058E+03	1.5973E+03	1.5866E+03	1.6585E+03	1.9016E+03	1.5939E+03	1.6810E+03	1.6376E+03
	Avg	1.2420E+03	1.9632E+03	1.7475E+03	1.6256E+03	1.6800E+03	1.7407E+03	2.0178E+03	1.8779E+03	1.7838E+03	1.7089E+03
	Std	4.7603E+01	5.3181E+01	1.8531E+01	1.8500E+01	4.7644E+01	6.1369E+01	6.3434E+01	1.0735E+02	8.4382E+01	2.9233E+01
F9	Min	7.2133E+03	4.5050E+04	4.7209E+03	1.0940E+03	1.8178E+04	2.5983E+04	3.7188E+04	2.8105E+04	3.0801E+04	3.9321E+03
	Avg	1.2625E+04	5.6636E+04	5.7731E+03	1.2340E+03	3.0780E+04	4.8143E+04	4.6902E+04	3.7309E+04	4.5731E+04	5.5413E+03
	Std	2.3640E+03	6.0940E+03	8.3553E+02	6.1096E+01	5.0756E+03	1.2048E+04	9.3195E+03	5.1849E+03	8.3157E+03	1.0870E+03
F10	Min	1.3357E+04	2.9245E+04	3.0184E+04	3.0505E+04	2.5038E+04	2.1504E+04	2.4093E+04	3.0576E+04	2.8483E+04	3.0779E+04
	Avg	1.6596E+04	3.0963E+04	3.1489E+04	3.1924E+04	2.7913E+04	2.4188E+04	2.6292E+04	3.1856E+04	3.0372E+04	3.1878E+04
	Std	1.7439E+03	8.9864E+02	5.6387E+02	4.9753E+02	1.4451E+03	1.3578E+03	1.3889E+03	6.1024E+02	1.0499E+03	5.5089E+02
F11	Min	3.9943E+03	3.1428E+04	4.4598E+04	5.5321E+03	3.3121E+04	2.8648E+04	4.1631E+04	6.1043E+04	5.3119E+04	1.0392E+04
	Avg	6.2596E+03	5.7146E+04	6.8703E+04	1.0474E+04	6.3619E+04	4.0656E+04	6.4624E+04	8.6596E+04	7.9376E+04	1.3902E+04
	Std	1.5578E+03	1.7066E+04	1.0817E+04	4.6092E+03	1.2551E+04	1.0258E+04	1.1699E+04	1.2763E+04	1.3072E+04	2.9036E+03
F12	Min	1.3640E+08	5.4902E+09	5.9297E+08	1.1991E+08	2.2790E+09	1.2504E+09	3.3246E+10	5.7041E+08	4.9899E+09	6.7650E+08
	Avg	4.3170E+08	7.8099E+09	7.4041E+08	1.6320E+08	3.9824E+09	3.1800E+09	5.2534E+10	1.2152E+09	9.4448E+09	9.6245E+08
	Std	2.2591E+08	1.4422E+09	9.0421E+07	2.8691E+07	1.0251E+09	9.5030E+08	9.4959E+09	3.3549E+08	3.2077E+09	1.6965E+08
F13	Min	9.2803E+04	2.6843E+08	3.2269E+06	2.5833E+06	1.5950E+07	2.4807E+07	4.6248E+09	2.3367E+06	8.7978E+07	1.4733E+07
	Avg	2.2390E+06	5.5600E+08	4.4902E+06	3.7350E+06	7.4561E+07	9.0213E+07	8.2509E+09	3.7264E+06	1.7287E+08	2.7605E+07
	Std	2.4319E+06	1.5582E+08	7.6293E+05	5.0930E+05	3.4196E+07	5.4687E+07	2.1722E+09	1.1087E+06	6.4125E+07	4.5739E+06
F14	Min	4.0105E+05	5.2954E+06	1.5545E+06	1.5788E+05	2.3004E+06	4.3213E+06	1.1975E+06	1.9909E+06	2.5488E+06	5.4056E+05
	Avg	1.6932E+06	9.7119E+06	2.8010E+06	2.3493E+05	6.4466E+06	8.1261E+06	1.0579E+07	5.0986E+06	6.0601E+06	9.1619E+05
	Std	7.8492E+05	2.9892E+06	8.5625E+05	3.9353E+04	2.6110E+06	3.3152E+06	3.4755E+06	1.8862E+06	2.9952E+06	2.8413E+05
F15	Min	3.4734E+04	3.3748E+07	1.5387E+05	6.9212E+05	8.8176E+05	2.1920E+06	6.8468E+08	1.7606E+05	6.0161E+05	3.1656E+06
	Avg	4.6612E+05	1.0337E+08	5.5898E+05	1.2336E+06	3.8104E+06	1.3268E+07	1.7343E+09	2.9760E+05	5.9231E+06	4.4307E+06
	Std	1.1442E+06	3.9464E+07	1.3209E+05	2.2519E+05	3.2396E+06	1.0764E+07	6.6276E+08	9.8005E+04	6.9425E+06	8.8448E+05
F16	Min	4.1281E+03	8.8534E+03	7.6751E+03	8.9412E+03	5.9077E+03	5.9375E+03	9.3330E+03	7.1688E+03	5.8483E+03	9.3256E+03
	Avg	5.6924E+03	1.0055E+04	9.4591E+03	9.6610E+03	6.8669E+03	7.4960E+03	1.0744E+04	8.5873E+03	8.1491E+03	1.0038E+04
	Std	7.8352E+02	7.0579E+02	5.1428E+02	3.3490E+02	5.5586E+02	7.2857E+02	7.6227E+02	1.1172E+03	1.0138E+03	3.8629E+02
F17	Min	3.7788E+03	6.9158E+03	6.0730E+03	6.4897E+03	4.2964E+03	4.8831E+03	6.8952E+03	5.4728E+03	5.0293E+03	6.6322E+03
	Avg	4.5054E+03	7.7431E+03	6.6981E+03	6.8741E+03	5.2492E+03	5.7792E+03	1.3417E+04	6.4999E+03	6.3632E+03	7.1747E+03
	Std	4.0781E+02	4.0354E+02	2.8598E+02	2.1086E+02	5.5248E+02	5.3031E+02	6.9517E+03	7.4516E+02	5.5920E+02	3.3300E+02
F18	Min	1.0925E+06	4.9259E+06	2.1937E+06	3.3315E+05	2.7708E+06	2.4513E+06	3.5856E+06	3.2668E+06	2.6120E+06	1.1774E+06
	Avg	2.3426E+06	1.3942E+07	3.2124E+06	4.9734E+05	6.2284E+06	1.2184E+07	8.9785E+06	5.6643E+06	6.6408E+06	2.0508E+06
	Std	8.9811E+05	6.3408E+06	5.9478E+05	8.0681E+04	3.4122E+06	5.0085E+06	4.2263E+06	2.1963E+06	2.7825E+06	7.5870E+05
F19	Min	2.4023E+05	6.0881E+07	1.4126E+06	1.0785E+06	2.8469E+06	1.3087E+07	7.7001E+08	1.3164E+06	8.4730E+06	3.7073E+06
	Avg	7.5339E+05	1.1715E+08	2.1535E+06	1.5272E+06	7.4103E+06	4.3429E+07	1.8307E+09	3.0009E+06	2.6968E+07	6.0914E+06
	Std	5.0220E+05	4.1085E+07	4.4018E+05	2.8110E+05	3.3752E+06	1.6199E+07	7.9062E+08	1.2056E+06	2.0770E+07	1.4364E+06
F20	Min	3.9156E+03	6.1061E+03	6.4660E+03	6.6598E+03	4.6351E+03	4.9645E+03	5.0242E+03	5.4118E+03	5.7669E+03	7.0299E+03
	Avg	4.8251E+03	6.8759E+03	7.0705E+03	7.3678E+03	5.7175E+03	5.7028E+03	5.8759E+03	7.1540E+03	6.8189E+03	7.4293E+03
	Std	5.8893E+02	3.6591E+02	3.0125E+02	2.9720E+02	5.6439E+02	4.0259E+02	4.2830E+02	5.5726E+02	6.1089E+02	2.0292E+02
F21	Min	2.6567E+03	3.3687E+03	3.0868E+03	3.0863E+03	3.0809E+03	3.0401E+03	3.5779E+03	3.3493E+03	3.2106E+03	3.1795E+03
	Avg	2.7689E+03	3.4821E+03	3.2210E+03	3.1512E+03	3.1369E+03	3.2675E+03	3.7277E+03	3.4506E+03	3.3851E+03	3.2400E+03
	Std	7.0626E+01	6.6066E+01	4.0518E+01	3.0651E+01	4.4258E+01	9.0336E+01	1.0371E+02	5.4437E+01	1.0128E+02	2.5160E+01
F22	Min	1.5269E+04	3.1791E+04	3.3063E+04	3.2078E+04	2.7255E+04	2.4399E+04	2.6174E+04	3.3353E+04	2.9985E+04	3.2958E+04
	Avg	1.9051E+04	3.3389E+04	3.3787E+04	3.4378E+04	2.9760E+04	2.7185E+04	2.8601E+04	3.3951E+04	3.1737E+04	3.4209E+04
	Std	1.3143E+03	7.5665E+02	5.0075E+02	7.3127E+02	1.2967E+03	1.2953E+03	1.8895E+03	4.1926E+02	1.1081E+03	4.6692E+02
F23	Min	3.1954E+03	3.8458E+03	3.6697E+03	3.6215E+03	3.6175E+03	3.6389E+03	4.3409E+03	3.8881E+03	3.8196E+03	3.7310E+03
	Avg	3.3197E+03	3.9925E+03	3.7420E+03	3.6853E+03	3.7039E+03	3.7629E+03	4.5566E+03	3.9704E+03	3.9312E+03	3.7727E+03
	Std	6.1616E+01	8.0157E+01	4.2501E+01	2.7166E+01	6.6148E+01	9.2376E+01	1.1630E+02	4.2048E+01	6.6271E+01	2.7552E+01
F24	Min	3.6240E+03	4.3568E+03	4.0639E+03	4.0889E+03	4.1233E+03	4.2143E+03	5.4314E+03	4.4006E+03	4.4267E+03	4.1856E+03
	Avg	3.7585E+03	4.5346E+03	4.1916E+03	4.1292E+03	4.2532E+03	4.4389E+03	5.7976E+03	4.5467E+03	4.6507E+03	4.2297E+03
	Std	6.9169E+01	1.1133E+02	5.2393E+01	2.2525E+01	8.0876E+01	1.5046E+02	2.2157E+02	8.0822E+01	1.3645E+02	2.5617E+01
F25	Min	3.8460E+03	5.5350E+03	4.0567E+03	3.5329E+03	6.0108E+03	4.6225E+03	1.1907E+04	4.5058E+03	7.9042E+03	3.8986E+03
	Avg	4.1238E+03	6.4587E+03	4.1849E+03	3.5803E+03	6.9994E+03	5.8749E+03	1.3328E+04	4.9184E+03	1.0029E+04	4.0627E+03
	Std	2.4718E+02	5.3562E+02	7.8966E+01	2.5077E+01	5.8032E+02	5.9102E+02	7.7631E+02	3.0436E+02	1.6648E+03	9.1138E+01
F26	Min	9.4230E+03	1.7353E+04	1.3225E+04	1.3570E+04	1.5339E+04	1.5208E+04	2.9783E+04	1.7129E+04	1.6330E+04	1.4502E+04
	Avg	1.0831E+04	1.8997E+04	1.4726E+04	1.4120E+04	1.6693E+04	1.7419E+04	3.2670E+04	1.8403E+04	1.9663E+04	1.5235E+04
	Std	7.3066E+02	9.5578E+02	5.4524E+02	2.1975E+02	1.0180E+03	1.0941E+03	1.9698E+03	8.5040E+02	1.9863E+03	2.5119E+02
F27	Min	3.5825E+03	4.0549E+03	3.5913E+03	3.4127E+03	3.8723E+03	3.9077E+03	4.6917E+03	3.6366E+03	3.8258E+03	3.7120E+03
	Avg	3.7572E+03	4.3132E+03	3.7063E+03	3.4367E+03	4.0707E+03	4.1537E+03	5.5338E+03	3.9230E+03	3.9931E+03	3.8002E+03
	Std	1.2996E+02	1.6402E+02	5.3263E+01	1.9274E+01	1.1801E+02	1.4724E+02	3.2406E+02	1.5311E+02	1.5937E+02	7.2320E+01
F28	Min	3.9203E+03	6.1113E+03	4.4819E+03	3.5550E+03	8.4222E+03	6.5943E+03	1.6301E+04	5.4895E+03	1.0378E+04	4.0913E+03
	Avg	4.7821E+03	8.3407E+03	4.7773E+03	3.6012E+03	1.0020E+04	8.7911E+03	1.8571E+04	6.5139E+03	1.4972E+04	4.5426E+03
	Std	5.7104E+02	1.5165E+03	1.5600E+02	2.1743E+01	8.4359E+02	1.2104E+03	1.0899E+03	7.4611E+02	3.1889E+03	2.8712E+02
F29	Min	6.5055E+03	9.6161E+03	9.0317E+03	8.3586E+03	7.2943E+03	7.9667E+03	1.2771E+04	8.2952E+03	8.1711E+03	8.4575E+03
	Avg	7.3822E+03	1.0948E+04	9.7329E+03	8.8513E+03	8.3068E+03	9.4405E+03	1.5266E+04	9.6429E+03	9.4178E+03	9.4710E+03
	Std	4.9021E+02	8.0456E+02	2.9988E+02	2.6875E+02	5.2810E+02	9.0092E+02	1.8364E+03	6.0010E+02	6.7016E+02	4.0765E+02
F30	Min	5.7096E+06	2.2498E+08	2.1022E+07	3.5636E+06	6.1952E+07	1.4066E+08	1.7071E+09	2.1606E+07	6.9711E+07	1.3821E+07
	Avg	1.6084E+07	4.7540E+08	2.6953E+07	4.9086E+06	1.2885E+08	3.3376E+08	5.6914E+09	4.5601E+07	2.0070E+08	2.2466E+07
	Std	7.3277E+06	1.7588E+08	4.4166E+06	8.0210E+05	4.7327E+07	1.1519E+08	2.5080E+09	1.2660E+07	1.3212E+08	6.3143E+06
